# Clinicopathological Study of Oncocytomas of Head and Neck Region: A Systematic Review

**DOI:** 10.1111/jop.70022

**Published:** 2025-08-06

**Authors:** João Paulo Gonçalves de Paiva, Laura Borges Kirschnick, Daniela Giraldo Roldán, Manoela Domingues Martins, Alan Roger Santos‐Silva, Ciro Dantas Soares, Jacks Jorge

**Affiliations:** ^1^ Departamento de Diagnóstico Oral, Faculdade de Odontologia de Piracicaba Universidade Estadual de Campinas São Paulo Brazil; ^2^ Private Pathology Service Getúlio Sales Diagnósticos, GSD Natal Rio Grande do Norte Brazil

**Keywords:** head and neck neoplasms, oxyphil cells, oxyphilic adenoma, salivary gland neoplasms

## Abstract

**Background:**

Salivary gland oncocytomas are infrequent benign salivary gland tumors with few reported cases.

**Aims:**

This study aimed to systematically review case reports and case series studies on oncocytomas in the head and neck region.

**Materials & Methods:**

Electronic searches were performed in PubMed, Scopus, Web of Science, Embase, and LILACS databases. The risk of bias was assessed using the Joanna Briggs Institute—University of Adelaide tool for case reports and case series.

**Results:**

A total of 99 studies (145 cases) were included. Oncocytomas predominantly affected women, typically presenting as solitary, asymptomatic parotid masses in patients over 51 years of age. Some cases reported multiple or bilateral tumors, occasionally associated with other salivary gland lesions. Histologically, the tumors were primarily composed of eosinophilic oncocytes with minimal pleomorphism, arranged in diverse architectural patterns. Immunohistochemical analysis revealed positivity for PTAH, antimitochondrial antigen, CK5/6, CK8/18, CK10/13, CK19, EMA, along with a low Ki67 index, while being negative for S100 and actin. Surgical excision was the primary treatment, with rare instances of recurrence.

**Conclusion:**

Oncocytoma is a rare, benign neoplasm that most commonly arises in the parotid gland, with a predilection for female patients. Complete surgical excision constitutes the standard treatment and is associated with an excellent prognosis.

## 1 Introduction

Salivary gland oncocytoma is a rare benign neoplasm, accounting for approximately 1.5% of all salivary gland tumors. It belongs to the spectrum of oncocytic salivary gland lesions, which encompasses nodular oncocytic hyperplasia and oncocytic carcinoma (the latter recently reclassified as an emerging entity in the latest World Health Organization classification of head and neck tumors) [1]. Oncocytomas typically present as solitary parotid masses in elderly patients, with no sex predilection [2]. However, they may occasionally manifest as multiple (sometimes bilateral) lesions and can be associated with other salivary gland lesions, including nodular oncocytic hyperplasia and Warthin tumor [3, 4, 5].

Histologically, oncocytomas are predominantly composed of epithelial cells exhibiting eosinophilic granular cytoplasm (oncocytes), typically arranged in solid sheets, islands, nests, trabeculae, cords, and duct‐like structures [6, 7]. Although this represents the classic morphological pattern, focal or, less commonly, diffuse clear cell changes may occur due to intracytoplasmic glycogen accumulation [8]. Immunohistochemically, these tumors typically exhibit cytoplasmic antimitochondrial antigen expression, p63 positivity in basal cells, and a low Ki67 proliferation index [2, 9]. Diagnosis is supported by histochemical stains, particularly phosphotungstic acid‐hematoxylin (PTAH), which highlights the tumor's abundant mitochondria [2]. For the clear cell variant, periodic acid–Schiff (PAS) staining confirms intracytoplasmic glycogen accumulation, thereby serving as an important diagnostic tool [8].

The standard treatment for salivary gland oncocytomas consists of complete surgical excision, which is typically curative [10]. The prognosis of oncocytomas is favorable, with low recurrence rates; although rare, malignant transformation into oncocytic carcinoma has been reported [11].

While previous systematic review have focused exclusively on salivary gland oncocytomas, the current study adopts a more comprehensive methodological framework [12]. Our search strategy incorporates additional databases, including LILACS to capture Spanish‐language literature, and extends the anatomical scope to encompass oncocytomas originating from both salivary and seromucous glands throughout the upper respiratory tract. Due to the limited number of reported cases, there remains a lack of comprehensive understanding regarding the features of oncocytomas in the head and neck region. To consolidate the existing literature on the topic, we conducted a systematic review addressing the clinical question: “What are the clinicopathological characteristics, immunohistochemical profile, preferred treatment approach, and recurrence frequency associated with salivary and seromucous glands oncocytoma?”

## 2 Methods

### 2.1 Eligibility Criteria

Our systematic review included case reports and case series published in English and Spanish that documented oncocytomas affecting major and minor salivary glands, as well as seromucous glands of the upper respiratory tract (including paranasal sinuses, pharynx, and larynx). To ensure diagnostic accuracy, we selected only studies that provided either high‐quality microscopic images or detailed histopathological descriptions confirming an oncocytoma diagnosis. We excluded studies with inadequate clinicopathological information, review articles, conference abstracts, retrospective studies lacking individual case data, and reports with inconclusive diagnostic findings.

### 2.2 Information Sources

This systematic review adhered to the Preferred Reporting Items for Systematic Reviews and Meta‐analyses (PRISMA) Statement [13] and was registered on the International Prospective Register of Systematic Reviews (PROSPERO) under the protocol number CRD42021241928.

Our systematic search strategy was implemented in two phases. An initial comprehensive search of MEDLINE/PubMed, Scopus, Web of Science, Embase, and LILACS databases was conducted in April 2024 without publication date restrictions; followed by an updated search in March 2025 to capture newly available studies. Additional searches were conducted in the grey‐literature databases ProQuest and Google Scholar, along with manual searches of reference lists from included articles to identify publications eventually missed by electronic searches. The complete search strategy for all databases is provided in the Appendix S1.

### 2.3 PECOS Framework

This systematic review was structured according to the PECOS framework, with the population (P) defined as patients of any age. The exposure (E) consisted of patients diagnosed with oncocytoma. Comparator (C) was not applicable due to the descriptive study design. The outcomes (O) assessed comprised clinical and demographic characteristics, histopathological features, immunohistochemical profiles, histochemical staining patterns, and imaging characteristics. We considered study design (S) to include case reports, case series, and cohort studies.

### 2.4 Selection Process

Two independent reviewers (J.P.G.P. and L.B.K.) screened all titles and abstracts retrieved through the initial search. Studies meeting the predefined eligibility criteria at the title/abstract stage underwent full‐text evaluation. The same reviewers then assessed these selected studies in full‐text format, including those identified through manual reference searches. Disagreements between reviewers were resolved through consensus‐based discussion, with unresolved cases arbitrated by a third author (D.G.R.).

### 2.5 Data Collection Process and Data Items

Two authors (J.P.G.P. and L.B.K.) independently extracted data from all included studies using Microsoft Excel (version 2203). The dataset included comprehensive study details: author names, publication year, country, study design, number of oncocytoma cases, patient demographics (sex and age), anatomic location, clinical presentation, symptoms, lesion number (solitary/multiple), laterality (unilateral/bilateral), imaging modalities and findings, radiotherapy history (yes/no. If yes, how long ago), management approach, association with other salivary gland lesions (yes/no, If yes, specific lesion), association with systemic disease or syndrome (yes/no. If yes, specific condition), recurrence (yes/no. If yes, time since treatment), follow‐up (months and status), histopathological characteristics, and immunohistochemical/special stain profiles. Discrepancies between reviewers were resolved through initial discussion, with unresolved cases referred to a third author (D.G.R.) for arbitration.

### 2.6 Study Risk of Bias Assessment

The methodological quality and risk of bias of included studies were evaluated using the Joanna Briggs Institute (JBI) critical appraisal tools for case reports and case series [14]. Each appraisal item was independently assessed by two authors, as recommended, and classified as “yes”, “no,” “unclear,” or “not applicable.” Studies were categorized by overall risk of bias based on the percentage of “yes” responses: studies scoring > 70% were classified as low risk, those scoring 50%–70% as moderate risk, and studies scoring < 50% as high risk of bias.

### 2.7 Effect Measures

The collected data were analyzed descriptively and presented as both absolute and relative frequencies (percentages). For numerical variables, measures of central tendency and dispersion were reported, including means with their standard deviations (SDs) and/or median values when applicable.

### 2.8 Synthesis Methods

The analyzed variables were categorized into three analytical categories: (1) clinical and demographic characteristics, (2) histopathological features (incorporating immunohistochemical and histochemical profiles), and (3) imaging findings. When studies failed to explicitly report whether specific variables were present or absent, these were recorded as not available (NA).

## 3 Results

### 3.1 Study Characteristics

**FIGURE 1:**
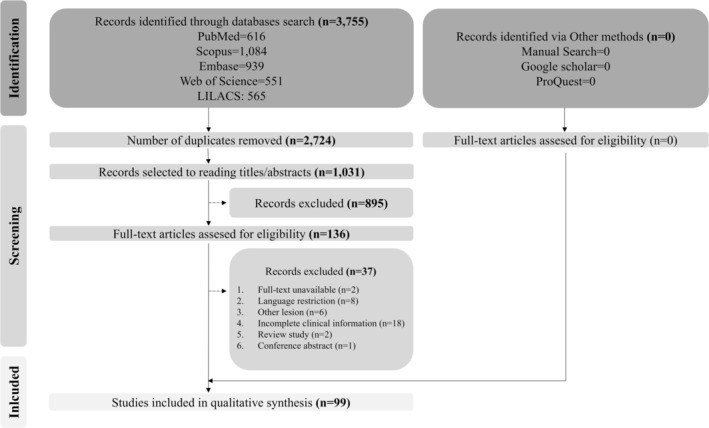
Selection process flowchart for the included studies in this systematic review.

The full dataset for all included oncocytoma cases is provided in Appendix S2. This systematic review incorporated 99 articles (86 case reports and 13 case series), encompassing 145 published cases. The publications spanned from 1948 to 2023, with the majority originating from the United States of America (*n* = 38), Japan (*n* = 13), and India (*n* = 10). Figure 1 illustrates the PRISMA flowchart detailing the study selection process.

### 3.2 Clinical and Epidemiological Features

As detailed in Table 1 and Appendix S2, our analysis revealed a slight female predominance among salivary and seromucous oncocytoma cases, with 74 female patients (51.40%) compared to 70 male patients (48.60%), resulting in a female‐to‐male ratio of 1.07:1. The age distribution showed a wide range, spanning from 5 to 89 years. The mean patient age was 58.68 years (SD ±16.75 years), with a median age of 62 years. Age distribution by decade demonstrated the highest prevalence in the 61–70 year group (*n* = 41/28.50%), followed by 71–80 years (*n* = 36/25%), and 51–60 years (*n* = 25/17.40%).

**TABLE 1:** Clinical and demographic characteristics, treatment, and prognostic features of oncocytoma cases included in this systematic review.

Variables	*n* (%)
Sex (*n* = 144)	
Female	74 (51.40)
Male	70 (48.60)
Age (*n* = 144) (years)	
0–10	3 (2.01)
11–20	3 (2.01)
21–30	3 (2.01)
31–40	9 (6.30)
41–50	20 (13.90)
51–60	25 (17.40)
61–70	41 (28.50)
71–80	36 (25.00)
81–90	4 (2.80)
Mean age (standard deviation)	58.68 (16.75) years
Median age	62 years
Male mean age (standard deviation)	58.10 (16.10) years
Male median age	61 years
Female mean age (standard deviation)	59.1 (17.3) years
Female median age	63 years
	Range 5–89 years
Anatomic location (*n* = 145)	
Parotid gland	94 (64.83)
Submandibular gland	12 (8.28)
Nasal cavity	6 (4.14)
Palate	5 (3.45)
Nasal septum	4 (2.76)
Buccal mucosa	4 (2.76)
Nasopharynx	3 (2.07)
Larynx	2 (1.38)
Maxillary sinus	2 (1.38)
Multiple sites	2 (1.38)
Tongue	2 (1.38)
Alveolar crest	1 (0.69)
Cheek	1 (0.69)
Ethmoid sinus	1 (0.69)
Parapharyngeal space	1 (0.69)
Retromandibular region	1 (0.69)
Clinical presentation of the lesion (*n* = 128)	
Nodular mass	128 (100.00)
Number of lesions (*n* = 143)	
Solitary	134 (93.71)
Multiple	9 (6.29)
Laterality (*n* = 144)	
Unilateral	138 (95.83)
Bilateral	6 (4.17)
Symptoms (*n* = 117)	
No	77 (65.81)
Yes	40 (34.19)
Pain	15 (37.50)
Epistaxis	6 (15.00)
Nasal congestion	5 (12.50)
Bleeding	3 (7.50)
Discomfort	3 (7.50)
Irritation	3 (7.50)
Rhinorrhea	3 (7.50)
Facial weakness	2 (5.00)
Hoarseness	2 (5.00)
Paresthesia	2 (5.00)
Deafness	1 (2.50)
Dysphagia	1 (2.50)
Facial palsy	1 (2.50)
Headache	1 (2.50)
Hearing loss	1 (2.50)
Itching	1 (2.50)
Odynophagia	1 (2.50)
Pressure sensation	1 (2.50)
Recurrent sinus infection	1 (2.50)
Tenderness	1 (2.50)
Transient ischemic attack	1 (2.50)
Radiotherapy history *(n* = 56)	
No	55 (98.21)
Yes	1 (1.79)
Association with other salivary gland lesions (*n* = 107)	
No	98 (91.59)
Oncocytic hyperplasia	4 (3.74)
Oncocytic nodular hyperplasia	3 (2.80)
Oncocytic nodular hyperplasia and oncocytic cysts	1 (0.93)
Warthin tumor	1 (0.93)
Disease or syndrome association (*n* = 84)	
No	74 (88.10)
Birt–Hogg–Dubé syndrome	2 (2.38)
Hairy cell leukemia, hypertension, and rheumatoid arthritis	1 (1.19)
HCV infection and non‐Hodgkin lymphoma	1 (1.19)
Hypertension	1 (1.19)
Immune thrombocytopenic purpura and hepatitis B	1 (1.19)
Multiple endocrine neoplasia 2B syndrome and neurofibromatosis type I	1 (1.19)
Sarcoidosis, osteoarthritis, asthma, gastroesophageal reflux	1 (1.19)
Treatment (*n* = 132)	
Surgery	126 (95.45)
Surgery and radiotherapy	2 (1.52)
Conservatively follow‐up	1 (0.76)
No treatment	1 (0.76)
Surgery and iodine‐125 therapy	1 (0.76)
Surgery and iodine‐131 therapy	1 (0.76)
Surgery and neck dissection	1 (0.76)
Surgery and selective embolization	1 (0.76)
Recurrence (*n* = 106)	
No	98 (92.45)
Yes	6 (5.66)
No complete surgical removal	2 (1.89)
Status (*n* = 85)	
Alive free of disease	84 (98.84)
Dead without disease	1 (1.16)
Mean follow‐up time (standard deviation)	36.6 (46.8) months
Median follow‐up time	24 months
	Range 2–263 months

Abbreviation: HCV, hepatitis C virus.

The oncocytoma cases involved 16 different anatomical sites, with the parotid gland being the predominant location (*n* = 94/66.83%). The submandibular gland was the second most frequent site (*n* = 12/8.28%). Among the 128 cases documenting clinical presentation, all presented as nodular masses (100%). Solitary lesions (*n* = 134/93.71%) were markedly more common than multiple lesions (*n* = 9/6.29%), with a 15:1 ratio. Similarly, unilateral involvement (*n* = 138/95.83%) strongly predominated over bilateral cases (*n* = 6/4.14%), demonstrating a 23:1 ratio.

Symptom data were available for 117 cases, with most patients (*n* = 77/65.81%) being asymptomatic. Among the 40 symptomatic cases (34.19%), we identified 21 distinct clinical manifestations. Pain (*n* = 15/37.50%) and epistaxis (*n* = 6/15%) were the most frequently reported symptoms, as detailed in Table 1 and Appendix S2.

The majority of patients (*n* = 55/98.21%) had no history of radiotherapy exposure. A single case (1.79%) reported childhood radiotherapy for acne treatment. Our analysis identified 10 cases where oncocytomas coexisted with other salivary gland lesions, most commonly oncocytic hyperplasia (*n* = 4/3.70%). Additionally, eight cases demonstrated associations with systemic conditions, with Birt–Hogg–Dubé (BHD) syndrome (*n* = 2/2.35%) being the most frequently observed syndromic association (Table 1, Appendix S2).

Primary surgical excision alone was performed in 126 patients (95.5%), demonstrating curative efficacy in 98 cases (92.5%). Recurrences occurred exclusively in parotid gland tumors (*n* = 6/5.7%), while two cases (1.9%) represented persistent disease due to incomplete resection (Table 1, Appendix S2).

Follow‐up data (range: 2–263 months; mean 36.6 months; median 24 months; SD ±46.8 months) from 86 patients revealed an excellent prognosis, with only one death (1.2%) attributable to acute myocardial infarction rather than oncocytoma progression (Table 1, Appendix S2).

### 3.3 Imaging Exams

Comprehensive imaging findings for 37 patients are detailed in Table 2 and Appendix S2. Computed tomography (CT) was the predominant imaging modality (*n* = 32/86.48%), though multiple techniques were often employed. Tumor diameters ranged from 6 mm to 120 mm (mean 30.89 mm; median 24.50 mm; SD ±23.64 mm), with most lesions appearing homogeneous on CT, although heterogeneous patterns were observed in some cases. Salivary gland tumors typically demonstrated well‐circumscribed margins without tissue infiltration, though larger lesions caused displacement of adjacent structures. In contrast, some sinonasal lesions exhibited aggressive features, including bone erosion.

**TABLE 2:** Imaging features of oncocytoma cases included in this systematic review.

Location	CT	MRI	Destruction/erosion of adjacent tissues	Lesion diameter	Delimitation
Parotid gland	Heterogeneous mass (*n* = 5) Homogeneous mass (*n* = 12)	Heterogeneous enhancement (*n* = 3) Hypointense mass (*n* = 6) Hypointense on T1 and hyperintense on T2 (*n* = 5)	No (*n* = 22)	Range: 15–120 mm Mean: 33.19 mm (SD: 24.36 mm) Median: 25 mm	Well‐defined (*n* = 22)
Submandibular gland	Heterogeneous mass (*n* = 2) Homogeneous mass (*n* = 4)	Not performed	No (*n* = 6)	Range: 13–100 mm Mean: 37.83 (SD: 33.34) Median: 24 mm	Well‐defined (*n* = 6)
Upper respiratory tract	Heterogeneous mass (*n* = 1) Homogeneous mass (*n* = 5)	Heterogeneous enhancement (*n* = 1)	Yes (*n* = 4) No (*n* = 2)	Range: 17–50 mm Mean: 30.66 mm (SD: 17.21 mm) Median: 25 mm	Well‐defined (*n* = 2) Ill‐defined (*n* = 2)
Oral cavity	Homogeneous mass (*n* = 2)	Not performed	No (*n* = 2)	Range: 15–26 mm Mean: 20.5 mm Median: 7.77 mm	Well‐defined (*n* = 2)
All cases	Homogeneous mass (*n* = 23) Heterogeneous mass (*n* = 8)	Heterogeneous enhancement mass (*n* = 9) Hypointense mass (*n* = 1)	No (*n* = 32) Yes (*n* = 4)	Range: 6–120 mm Mean 30.89 mm (SD: 23.64 mm) Median: 24.50 mm	Well‐defined (*n* = 32) Ill‐defined (*n* = 2)

Abbreviations: CT, computed tomography; MRI, magnetic resonance image; SD, standard deviation.

### 3.4 Histopathological Features

**FIGURE 2:**
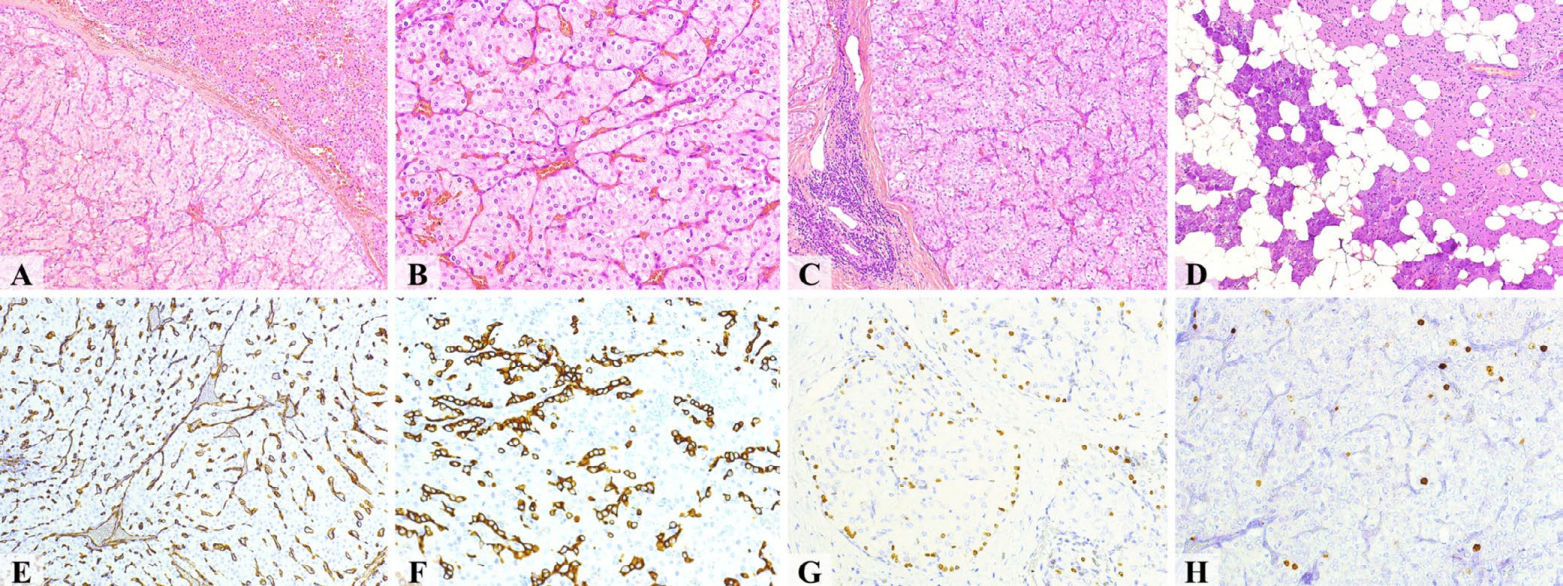
Histopathological and immunohistochemical features of an oncocytoma case from the author's files. (A) The lesion displays a multinodular architecture with alternating areas of large, clear oncocytes and oncocytes with intensely eosinophilic cytoplasm. (B) Oncocytes typically show finely granular eosinophilic cytoplasm with centrally located, round vesicular nuclei containing prominent nucleoli, supported by a delicate vascular network within sparse stroma. (C) Remnant of salivary gland tissue demonstrates focal areas of oncocytic hyperplasia. (D) Adjacent to large tumor nodules, occasional lymphoid aggregates are observed. (E) The CD34 immunohistochemistry highlights the stromal capillaries. (F) CK7 shows distinct membranous positivity in oncocytes. (G) The p63 immunostaining identifies basal cells surrounding large oncocyte nodules. (H) A markedly low Ki67 positivity in oncocytes.

Appendix S3 and Table 3 detail the histopathological features of 145 cases of oncocytoma. The tumors were primarily composed of eosinophilic oncocytes (*n* = 122/84.14%); though a subset (*n* = 20/13.79%) exhibited a mixed population of clear and eosinophilic oncocytes. Pure clear cell oncocytomas were rare (*n* = 3/2.07%). Focal pleomorphism was infrequent (*n* = 4/2.76%). The predominant nuclear feature was vesicular nuclei with prominent nucleoli (*n* = 123/84.83%). Among 139 cases with documented architecture, most tumors displayed multiple growth patterns. The most common were solid (*n* = 65/46.76%) and sheet‐like (*n* = 54/38.85%). Cystic spaces were identified in 70 of 109 cases (64.22%), while lymphoid stroma was observed in 18 of 41 (43.90%) cases. Figure 2 illustrates a parotid oncocytoma from the author's files.

**TABLE 3:** Histopathological characteristics of oncocytoma cases included in this systematic review.

Histopathological feature	*n* (%)
Lobulation (*n* = 92)	
Unilobulated	48 (48.86)
Multilobulated	44 (51.14)
Oncocytes appearance (*n* = 145)	
Eosinophilic oncocytes	122 (84.14)
Eosinophilic and clear oncocytes	20 (13.79)
Clear oncocytes	3 (2.07)
Pleomorphism (*n* = 145)	
Yes	4 (2.76)
No	141 (97.24)
Nuclear characteristics (*n* = 145)	
Vesicular nuclei	123 (84.83)
Hyperchromatic condensed nuclei	14 (9.66)
Vesicular and hyperchromatic condensed nuclei	8 (5.52)
Mitotic figures (*n* = 139)	
Yes	2 (1.44)
No	137 (98.56)
Arrangement pattern (*n* = 139)	
Solid	65 (46.76)
Sheets	54 (38.85)
Duct‐like structures	47 (33.81)
Clusters	37 (26.62)
Nests	37 (26.62)
Trabecular	28 (20.14)
Cords	19 (13.67)
Tubular	13 (9.35)
Alveolar	9 (6.47)
Cystic	9 (6.47)
Nodular	9 (6.47)
Islands	6 (4.32)
Columnar	5 (3.60)
Papillary	3 (2.16)
Organoid	3 (2.16)
Cystic spaces (*n* = 109)	
Yes	70 (64.22)
No	39 (35.78)
Lymphoid stroma (*n* = 41)	
Yes	18 (43.90)
No	23 (56.10)

### 3.5 Immunohistochemical Markers and Special Stains

Table 4 summarizes the immunohistochemical and histochemical profiles of oncocytomas. The tumors consistently showed positivity for PTAH and immunoreactivity to antimitochondrial antigen. Epithelial markers were frequently expressed, including AE1/AE3, CK5/6, CK8/18, CK10/13, CK19, and EMA. p63 immunostaining highlighted the myoepithelial layer surrounding oncocytic nests. Oncocytoma cases exhibited characteristically low Ki‐67 proliferation indices. Common negative markers included S100, TTF1, CEA, thyroglobulin, chromogranin, and actin (Figure 2).

**TABLE 4:** Immunohistochemical profile and special staining patterns in oncocytoma cases included in this systematic review.

Author/year	Immunohistochemical profile	Special stain
Anzalone et al. (2019)	p63 (+) myoepithelial cells	PTAH (+)
Araki and Sakaguchi (2004)	Not informed	PTAH (+)
Broekhuizen et al. (2011)	AE1/AE3 (+), CK7/8 (+), CK34 (+), Be 12 (+), CK5/6 (+), Ki67 (low). TTF1 (−), neuroendocrine markers (−), chromogranin A (−), synaptophysin (−), actin 1A4 (−), CEA (−), S100 (−)	Not informed
Camara et al. (2005)	Not informed	PAS (+)
Chau and Radden (1986)	Not informed	PTAH (+). PAS (−), alcian blue (−), mucicarmine (−)
Chui et al. (1985)	Not informed	PTAH (+)
Cohen and Batsakis (1968)	Not informed	PTAH (+)
Comin et al. (1997)	Not informed	PTAH (+)
Damm et al. (1989)	Not informed	PTAH (+)
Hamada et al. (2018)	Ki67 (low)	Not informed
Hamdan et al. (2002)	Cytokeratin (+), vimentin (−), Actin (−), desmin (−), S100 (−), thyroglobulin (−), alpha‐fetoprotein (−)	Not informed
Holmes et al. (1998)	PSA (+), PAP (+)	Not informed
Ito et al. (2000)	Ki67 (3.4%) and Ki67 (3.2%), in one case each	Not informed
Jo et al. (2010)	c‐kit (+), p53 (+), ki67 (< 1%). EGFR (−), HER‐2 (−)	Not informed
	p53 (+). EGFR (−), HER‐2 (−)	Not informed
	p53 (+). EGFR (−), HER‐2 (−)	Not informed
	c‐kit (+), p53 (+), ki67 (< 1%)	Not informed
	p53 (+), ki67 (< 1%). EGFR (−), HER‐2 (−)	Not informed
	c‐kit (+), p53 (+), ki67 (< 1%). EGFR (−), HER‐2 (−)	Not informed
Kanazawa et al. (2000)	Not informed	PTAH (+)
Lopez et al. (2013)	S100 (+), ki67 (< 5%)	Not informed
McLoughlin et al. (1994)	Not informed	PTAH (+)
Mercut et al. (2015)	Not informed	PTAH (+)
Miracco et al. (1986)	Cytokeratin (+)	Not informed
Palakshappa et al. (2014)	Not informed	PTAH (+)
Popovski et al. (2016)	Not informed	PTAH (+)
Skálová et al. (1999)^b^	Antimitochondrial antigen (+)	Not informed
	Antimitochondrial antigen (+)	Not informed
Stomeo et al. (2006)	Cytokeratin (+). CEA (−), S100 (−), SMA (−)	Not informed
Watanabe et al. (2011)	AE1/AE3 (+), ki67 (< 1%). S100 (−), CD68 (−), myoglobin (−)	Not informed
Yaku et al. (1985)	Not informed	PAS (+), alcian blue (−)
Yamazaki et al. (2018)	CK7 (+), CK5/6 (+), antimitochondrial antigen (+), p63 (+) myoepithelial cells, ki67 (low)	PAS (+)
Yoshihara et al. (1997)	Not informed	PTAH (+)
Zhou and Gao (2009)	CK5/6 (+), CK8/18 (+), CK10/13 (+), CK19 (+), EMA (+), ki67 (low)^a^	PTAH (+)

Abbreviations: CEA, carcinoembryonic antibody; EGFR, epithelial growth factor receptor; PAP, prostate acid phosphatase; PAS, periodic acid‐Schiff, PSA, prostatic specific antibody; PTAH, phosphotungstic acid hematoxylin.

^a^ Consistent data in 21 cases.

^b^ Consistent in two cases.

### 3.6 Determination of the Methodological Quality of the Included Studies

Critical appraisal of the 86 case reports included in this systematic review (Appendix S4) revealed that 55 studies (64%) were classified as low risk of bias, while 31 (36%) demonstrated moderate risk. The most frequent methodological limitations in case reports involved inadequate reporting of clinical timelines (78% of moderate‐risk studies), omission of adverse events (58%), and insufficient post‐intervention follow‐up details (52%). Among the 13 case series (Appendix S5), methodological quality was notably lower, with 10 studies (76.90%) categorized as moderate risk, and 3 achieving low risk classification. Case series exhibited more fundamental shortcomings, particularly unclear inclusion criteria, non‐consecutive participant inclusion, lack of statistical analysis, and inadequate outcome reporting. Appendix S6 contains the reference list of articles included in this systematic review that were not cited in the main text.

## 4 Discussion

This systematic review analyzed the clinicopathological characteristics of 145 oncocytomas arising in salivary and seromucous glands. Classified by the World Health Organization as a rare benign epithelial tumor, oncocytomas account for less than 1.5% of all salivary gland neoplasms [15]. The limited number of our study further underscores the rarity of oncocytomas compared to other systematic reviews of salivary gland tumors [16, 17]. These findings highlight the importance of carrying out more robust studies, such as systematic reviews, to better characterize the overall profile of oncocytoma.

Our systematic review identified a slight female predominance among oncocytoma patients, aligning with several previous reports [18, 19, 20]. However, the literature reveals conflicting data, with some studies reporting male predominance [10] while others document equal gender distribution [21, 22]. We observed an earlier peak prevalence occurring between the fifth and eighth decades of life, compared to the typically reported sixth to eighth decades in the literature [10, 21]. Notably, pediatric and adolescent cases were exceptionally rare among the included studies [10, 23, 24].

Oncocytomas are classically described as solitary nodular masses, though rare multifocal or bilateral presentations have been reported [3, 10, 18, 25]. Our review identified only four cases of bilateral multifocal oncocytomas [4, 5, 26, 27], and three cases of unilateral multifocal lesions [28, 29, 30], underscoring the exceptional rarity of these presentations. Accurate clinical differentiation is crucial, as multifocal oncocytomas must be distinguished from other oncocytic salivary gland lesions such as oncocytosis and oncocytic nodular hyperplasia. Oncocytosis demonstrates diffuse, unencapsulated oncocytic proliferation (predominantly in the parotid gland), while oncocytic nodular hyperplasia presents as multifocal unencapsulated growth. In contrast, oncocytomas are distinguished by their characteristic well‐defined capsule [3].

Oncocytomas are typically slow‐growing, asymptomatic tumors. However, a subset of patients may experience symptoms such as pain or epistaxis [31]. While these symptoms are not indicative of malignancy or perineural invasion, they may arise secondary to bone erosion caused by larger lesions [5]. Notably, epistaxis is a common clinical manifestation of space‐occupying sinonasal tumors and is frequently observed in oncocytomas involving these locations [32].

Oncocytomas may develop synchronously with other salivary gland lesions, particularly oncocytic hyperplasia and oncocytic nodular hyperplasia [5]. Synchronous ipsilateral salivary gland tumors are rare, accounting for less than 0.3% of all salivary gland neoplasms [33]. These associations have prompted investigations into oncocytoma pathogenesis, with emerging evidence suggesting a potential progression from non‐neoplastic oncocytic proliferations to neoplastic transformation, though this theory remains debated [3, 34, 35]. Notably, oncocytomas show a recognized association with BHD syndrome and are currently considered among its potential clinical manifestations [36, 37]. BHD syndrome, a rare autosomal dominant disorder caused by the folliculin gene alterations, provides important insights into oncocytoma biology. The occurrence of oncocytic tumors in BHD patients supports classifying oncocytomas as true neoplasms rather than hyperplastic processes [38].

Oncocytomas typically appear as well‐circumscribed masses with homogeneous enhancement on CT imaging [39, 40]. However, heterogeneous enhancement patterns may occur in some cases, potentially mimicking malignant tumors. CT characteristics can help differentiate oncocytomas from other benign salivary gland tumors. Warthin tumor typically demonstrates early‐phase enhancement that decreases in delayed phases, while pleomorphic adenomas exhibit minimal early enhancement with progressive delayed enhancement. In contrast, oncocytomas maintain homogeneous enhancement throughout both phases [39, 41]. Our systematic review revealed that paranasal oncocytomas frequently display aggressive imaging features, such as bone destruction and local invasion. Some authors suggested these sinonasal lesions may exhibit locally aggressive behavior akin to low‐grade malignancies, though this classification remains controversial in the literature [42, 43].

This systematic review found that most oncocytomas exhibited classic histopathological features, consisting entirely of eosinophilic oncocytes with vesicular nuclei and prominent nucleoli, absent significant pleomorphism or mitotic figures, and predominantly showing a solid growth pattern. These characteristics align with previously described diagnostic criteria [6, 10, 44]. Although clear cell changes have been suggested to occur more frequently in oncocytomas arising from oncocytic nodular hyperplasia, such features were uncommon in our study [3]. Pure clear cell oncocytomas are exceptionally uncommon in this study, substantiating the established rarity of this histological variant within the spectrum of oncocytic neoplasms. Our findings corroborate existing literature, including a case series study reporting only 1 clear cell oncocytoma among 21 cases [10]. Additionally, some tumors contained sparse lymphocytic infiltrates, which appeared as small, unorganized aggregates lacking germinal centers. This feature helps distinguish oncocytoma from the epithelial‐rich Warthin tumor, where lymphoid stroma is typically more prominent and organized [33].

The characteristic immunoprofile of oncocytoma demonstrates consistent positivity for antimitochondrial antigen and epithelial markers, while being negative for S100, SOX10, and SMA [2, 6, 10]. These immunophenotypic patterns support the hypothesized intercalated duct cell origin of these tumors [10]. The p63 antibody serves as a valuable diagnostic tool, highlighting basal layer cells and aiding differentiation from metastatic renal cell carcinoma and acinic cell carcinoma, as its positivity in the basal layer cells of oncocytoma and negativity in the other tumors [21, 45]. Mitochondrial markers, including PTAH staining and anti‐mitochondrial antigen immunohistochemistry, remain fundamental diagnostic adjuncts due to the tumor's abundant mitochondria [10, 46].

Surgical excision remains the gold‐standard treatment for oncocytoma and is typically curative [10, 46, 47]. Our findings support this approach, with only rare cases of postoperative recurrence reported. Notably, two patients in this study received adjuvant radiotherapy due to initial diagnostic uncertainty, though the therapeutic role of radiation in oncocytoma management remains unclear [46, 48]. While radiotherapy is non‐curative, some evidence suggests it may paradoxically increase recurrence risks, a concern potentially linked to radiation's debated role as an etiological factor for oncocytic lesions [24, 49]. Disease‐specific mortality was absent in our study, consistent with the overall excellent prognosis of oncocytoma [10]. However, given documented cases of malignant transformation to oncocytic carcinoma, long‐term clinical surveillance is recommended despite the tumor's predominantly benign behavior [10, 11].

This systematic review identified important limitations in existing literature on oncocytomas. Most available studies consist of case reports, which provide low scientific evidence due to their descriptive nature and small sample sizes. The rarity of this lesion has resulted in a scarcity of large case series or cohort studies, significantly restricting the scope of our analysis. Furthermore, we observed inconsistencies in study quality, with many case reports failing to follow standardized reporting guidelines or provide complete clinical information. To strengthen future research, we emphasize the need for case reports and case series studies to adhere to the CARE guidelines [14], and observational studies to comply with the STROBE statement [50]. Multicenter collaborative efforts would be particularly valuable to overcome current sample size limitations and generate more robust evidence regarding this uncommon tumor.

## 5 Conclusion

This study confirms that oncocytoma represents a rare benign salivary gland neoplasm, showing a predilection for the parotid gland in older patients. Clinically, these tumors typically manifest as solitary, asymptomatic masses. Histopathological examination reveals a predominant population of eosinophilic oncocytes, though focal or diffuse clear cell changes may occasionally occur. Complete surgical excision remains the treatment of choice, leading to cure in most cases, with minimal recurrence risk. Despite the generally favorable prognosis, long‐term clinical follow‐up is recommended to monitor potential rare outcomes.

## Author Contributions

**João Paulo Gonçalves de Paiva:** conceptualization, data curation, formal analysis, investigation, methodology, validation, writing – original draft. **Laura Borges Kirschnick:** conceptualization, data curation, formal analysis, investigation, methodology, validation, writing – review and editing. **Daniela Giraldo Roldán:** conceptualization, data curation, formal analysis, investigation, methodology, validation, writing – review and editing. **Manoela Domingues Martins:** conceptualization, data curation, formal analysis, investigation, methodology, validation, writing – review and editing. **Alan Roger Santos‐Silva:** conceptualization, data curation, formal analysis, investigation, methodology, validation, writing – review and editing. **Ciro Dantas Soares:** conceptualization, data curation, formal analysis, investigation, methodology, validation, writing – review and editing. **Jacks Jorge:** conceptualization, data curation, formal analysis, investigation, methodology, validation, supervision, writing – review and editing.

## Conflicts of Interest

The authors declare no conflicts of interest.

## Peer Review

The peer review history for this article is available at https://www.webofscience.com/api/gateway/wos/peer‐review/10.1111/jop.70022.

## Supporting information


**Appendix S1:** Full search strategies used in each database.

**Appendix S2:** Full clinicopathological information for the included cases in this systematic review.

**Appendix S3:** Histopathological features of oncocytoma cases included in this systematic review.

**Appendix S4:** Critical appraisal of case reports included in this systematic review.

**Appendix S5:** Critical appraisal of case series studies included in this systematic review.

**Appendix S6:** Reference list of studies included in this systematic review but not cited in the text.

## Data Availability

The data that supports the findings of this study are available in the Supporting Information of this article.
