# Supplementary material for: Clinicopathological Study of Oncocytomas of Head and Neck Region: A Systematic Review
Source: J Oral Pathol Med. 2025 Aug 6;54(8):635–46. doi: 10.1111/jop.70022 (PMC12419982; doi:10.1111/jop.70022)
Supplement: Supplementary file 1 — Appendix S1: Full search strategies used in each database. [file JOP-54-635-s005.docx]

| **PubMed**  **Appendix S1.** Full search strategies used in each database. | ("Oxyphilic Adenoma" OR "Oxyphilic adenomas" OR "Oncocytoma" OR "Oncocytomas" OR "Oncocytic adenoma" OR "Oncocytic adenomas" OR "Oxyphilic granular cell adenoma" ) AND ("Salivary glands" OR "Salivary gland" OR "Parotid gland" OR "Parotid glands" OR "Submandibular gland" OR "Submandibular glands" OR "Sublingual gland" OR "Sublingual glands" OR "Minor Salivary Gland" OR "Minor Salivary Glands" OR "Oral Cavity" OR "Mouth Cavity" OR "Mouth floor" OR "Sublingual region" OR "Sublingual regions" OR "Mouth mucosa" OR "Oral mucosa" OR "Buccal mucosa" OR Palates OR "Hard palate" OR "Hard Palates" OR "Palatine bone" OR Jaw OR Jaws OR Mandible OR Mandibles OR Maxilla OR Maxillas OR "Maxillary Bone" OR "Maxillary Bones" OR "Parotid region" OR Gnathic OR "Maxillary sinus" OR "Maxillary antrum" OR "Maxillary antrums" OR "Paranasal sinuses" OR "Paranasal sinus" OR "Nasal sinuses" OR "Nasal sinus" OR "Sinonasal tract" OR head OR Pharynx OR Hypopharynx OR Nasopharynx OR Oropharynx OR Pharyngeal OR Hypopharyngeal OR Nasopharyngeal OR Oropharyngeal) |
| --- | --- |
| **Scopus** | TITLE-ABS-KEY ("Oxyphilic Adenoma" OR "Oxyphilic adenomas" OR "Oncocytoma" OR "Oncocytomas" OR "Oncocytic adenoma" OR "Oncocytic adenomas" OR "Oxyphilic granular cell adenoma" ) AND TITLE-ABS-KEY ("Salivary glands" OR "Salivary gland" OR "Parotid gland" OR "Parotid glands" OR "Submandibular gland" OR "Submandibular glands" OR "Sublingual gland" OR "Sublingual glands" OR "Minor Salivary Gland" OR "Minor Salivary Glands" OR "Oral Cavity" OR "Mouth Cavity" OR "Mouth floor" OR "Sublingual region" OR "Sublingual regions" OR "Mouth mucosa" OR "Oral mucosa" OR "Buccal mucosa" OR Palates OR "Hard palate" OR "Hard Palates" OR "Palatine bone" OR Jaw OR Jaws OR Mandible OR Mandibles OR Maxilla OR Maxillas OR "Maxillary Bone" OR "Maxillary Bones" OR "Parotid region" OR Gnathic OR "Maxillary sinus" OR "Maxillary antrum" OR "Maxillary antrums" OR "Paranasal sinuses" OR "Paranasal sinus" OR "Nasal sinuses" OR "Nasal sinus" OR "Sinonasal tract" OR head OR Pharynx OR Hypopharynx OR Nasopharynx OR Oropharynx OR Pharyngeal OR Hypopharyngeal OR Nasopharyngeal OR Oropharyngeal) |
| **Embase** | ('Oxyphilic Adenoma' OR 'Oxyphilic adenomas' OR 'Oncocytoma' OR 'Oncocytomas' OR 'Oncocytic adenoma' OR 'Oncocytic adenomas' OR 'Oxyphilic granular cell adenoma' ) AND (‘Salivary glands' OR 'Salivary gland' OR 'Parotid gland' OR 'Parotid glands' OR 'Submandibular gland' OR 'Submandibular glands' OR 'Sublingual gland' OR 'Sublingual glands' OR 'Minor Salivary Gland' OR 'Minor Salivary Glands' OR 'Oral Cavity' OR 'Mouth Cavity' OR 'Mouth floor' OR 'Sublingual region' OR 'Sublingual regions' OR 'Mouth mucosa' OR 'Oral mucosa' OR 'Buccal mucosa' OR Palates OR 'Hard palate' OR 'Hard Palates' OR 'Palatine bone' OR Jaw OR Jaws OR Mandible OR Mandibles OR Maxilla OR Maxillas OR 'Maxillary Bone' OR 'Maxillary Bones' OR 'Parotid region' OR Gnathic OR 'Maxillary sinus' OR 'Maxillary antrum' OR 'Maxillary antrums' OR 'Paranasal sinuses' OR 'Paranasal sinus' OR 'Nasal sinuses' OR 'Nasal sinus' OR 'Sinonasal tract' OR head OR Pharynx OR Hypopharynx OR Nasopharynx OR Oropharynx OR Pharyngeal OR Hypopharyngeal OR Nasopharyngeal OR Oropharyngeal) |
| **Web of Science** | TS=(‘Oxyphilic Adenoma' OR 'Oxyphilic adenomas' OR 'Oncocytoma' OR 'Oncocytomas' OR 'Oncocytic adenoma' OR 'Oncocytic adenomas' OR 'Oxyphilic granular cell adenoma') AND TS=(‘Salivary glands' OR 'Salivary gland' OR 'Parotid gland' OR 'Parotid glands' OR 'Submandibular gland' OR 'Submandibular glands' OR 'Sublingual gland' OR 'Sublingual glands' OR 'Minor Salivary Gland' OR 'Minor Salivary Glands' OR 'Oral Cavity' OR 'Mouth Cavity' OR 'Mouth floor' OR 'Sublingual region' OR 'Sublingual regions' OR 'Mouth mucosa' OR 'Oral mucosa' OR 'Buccal mucosa' OR Palates OR 'Hard palate' OR 'Hard Palates' OR 'Palatine bone' OR Jaw OR Jaws OR Mandible OR Mandibles OR Maxilla OR Maxillas OR 'Maxillary Bone' OR 'Maxillary Bones' OR 'Parotid region' OR Gnathic OR 'Maxillary sinus' OR 'Maxillary antrum' OR 'Maxillary antrums' OR 'Paranasal sinuses' OR 'Paranasal sinus' OR 'Nasal sinuses' OR 'Nasal sinus' OR 'Sinonasal tract' OR head OR Pharynx OR Hypopharynx OR Nasopharynx OR Oropharynx OR Pharyngeal OR Hypopharyngeal OR Nasopharyngeal OR Oropharyngeal) |
| **LILACS** | ("Oxyphilic Adenoma" OR "Oxyphilic adenomas" OR "Oncocytoma" OR "Oncocytomas" OR "Oncocytic adenoma" OR "Oncocytic adenomas" OR "Oxyphilic granular cell adenoma" ) AND ("Salivary glands" OR "Salivary gland" OR "Parotid gland" OR "Parotid glands" OR "Submandibular gland" OR "Submandibular glands" OR "Sublingual gland" OR "Sublingual glands" OR "Minor Salivary Gland" OR "Minor Salivary Glands" OR "Oral Cavity" OR "Mouth Cavity" OR "Mouth floor" OR "Sublingual region" OR "Sublingual regions" OR "Mouth mucosa" OR "Oral mucosa" OR "Buccal mucosa" OR Palates OR "Hard palate" OR "Hard Palates" OR "Palatine bone" OR Jaw OR Jaws OR Mandible OR Mandibles OR Maxilla OR Maxillas OR "Maxillary Bone" OR "Maxillary Bones" OR "Parotid region" OR Gnathic OR "Maxillary sinus" OR "Maxillary antrum" OR "Maxillary antrums" OR "Paranasal sinuses" OR "Paranasal sinus" OR "Nasal sinuses" OR "Nasal sinus" OR "Sinonasal tract" OR head OR Pharynx OR Hypopharynx OR Nasopharynx OR Oropharynx OR Pharyngeal OR Hypopharyngeal OR Nasopharyngeal OR Oropharyngeal) |
