# Supplementary material for: Clinicopathological Study of Oncocytomas of Head and Neck Region: A Systematic Review
Source: J Oral Pathol Med. 2025 Aug 6;54(8):635–46. doi: 10.1111/jop.70022 (PMC12419982; doi:10.1111/jop.70022)
Supplement: Supplementary file 2 — Appendix S2: Full clinicopathological information for the included cases in this systematic review. [file JOP-54-635-s001.docx]

| **Author/Year**  **Appendix S2.** Full clinicopathological information for the included cases in this systematic review. | **Country** | **Study Design** | **N of cases** | **Sex** | **Age** | | **Site** | **Primary lesion** | **Symptomatology** | **Solitary or multiple** | **Unilateral or bilateral** | **Imaging exam** | **Recurrence** | **Management modality** | **RT history** | **Association with other lesions** | **Systemic disease or Syndrome Association** | **Follow-up Status** |
| --- | --- | --- | --- | --- | --- | --- | --- | --- | --- | --- | --- | --- | --- | --- | --- | --- | --- | --- |
| Adhikari et al. (2006) | Nepal | Case report | 1 case | F | 73 | Maxillary sinus | | NA | Pain+discomfort+epiphora | Solitary | Unilateral | CT: homogeneous opacity in maxillary antrum with expansion of walls. Well-encapsulated heterogenous mass occupying the right nasal cavity and maxillary antrum with partial bony destruction of the floor, roof, and medial walls. Lesion extending to the anterior ethmoidal sinus. Nasal septum was pushed to the opposite side | NA | Surgery | NA | No | NA | NA |
| Akhtar et al. (2016) | India | Case report | 1 case | M | 32 | Parotid gland | | Nodule | No | Solitary | Unilateral | CT: homogeneous hyperdense mass in superficial lobe of right parotid with regular contours. | NA | Surgery | No | No | NA | NA |
| Albers et al. (1993) | Belgium | Case report | 1 case | M | 70 | Nasopharynx | | Nodule | Bleeding | Solitary | Unilateral | CT: does not show any sign of invasive growth. MRI: no infiltration into the surrounding tissues and no sign of cervical lymphadenopathy | No | Surgery | NA | No | No | 12 months - FOD |
| Altman et al. (1998) | United States of America | Case report | 1 case | F | 67 | Nasal cavity | | Nodule | Recurrent sinus infections+ nasal congestion+ headaches+ rhinorrhea | Solitary | Unilateral | CT: 2.5x2.5 cm hyperdense mass involving the right inferior meatus and extending down to the floor of the nasal cavity | No | Surgery | Yes: in childhood for acne | No | No | 60 months - FOD |
| Anzalone et al. (2019) | United States of America | Case report | 1 case | F | 87 | Parotid gland | | Nodule | Transient ischemic attack | Solitary | Unilateral | CT: heterogeneous mass involving the superficial and deep lobe of left parotid containing both macroscopic and soft tissue elements. No evidence of erosion of the adjacent mandible. MRI: heterogenous T1 hyperintensity. Loss of the normal fat signal, which appears more il-defined and "hazy" | No | Surgery | NA | No | NA | 2 months -FOD |
| Araki and Sakaguchi (2004) | Japan | Case report | 1 case | M | 81 | Parotid gland | | Nodule | No | Solitary | Unilateral | CT: round and slightly enhanced. MRI: slightly and homogeneously enhanced in gadolinium-enhanced MRI. | No | Surgery | NA | Ipsilateral Warthin tumor | NA | 24 months - FOD |
| Askew et al. (1971) | United States of America | Case report | 1 case | F | 60 | Submandibular gland | | Nodule | No | Solitary | Unilateral | NA | NA | Surgery | NA | Oncocytic hyperplasia | NA | NA |
| Avila et al. (2019) | Argentina | Case serie | 5 cases | M | 26 | Parotid gland | | NA | NA | Solitary | Unilateral | NA | NA | Surgery | NA | NA | NA | NA |
|  |  |  |  | M | 64 | Parotid gland | | NA | NA | Solitary | Unilateral | NA | NA | Surgery | NA | NA | NA | NA |
|  |  |  |  | F | 55 | Parotid gland | | NA | NA | Solitary | Unilateral | NA | NA | Surgery | NA | NA | NA | NA |
|  |  |  |  | M | 76 | Parotid gland | | NA | NA | Solitary | Unilateral | NA | NA | Surgery | NA | NA | NA | NA |
|  |  |  |  | F | 45 | Parotid gland | | NA | NA | Solitary | Unilateral | NA | NA | Surgery | NA | NA | NA | NA |
| Banerjee et al. (1995) | United States of America | Case report | 1 case | M | 64 | Nasal cavity | | Nodule | Hearing loss | Solitary | Unilateral | CT: superficial lesion without evidence of bone erosion or extension into the parapharyngeal space | No | Surgery | NA | No | NA | 24 months - FOD |
| Barrese et al. (2010) | United States of America | Case report | 1 case | M | 73 | Parapharyngeal space | | Nodule | No | Solitary | Unilateral | CT: lesion with significant F-fluorodeoxyglucose uptake on positron emission tomography, and maximal standardized uptake value of 22.7. MRI: separation from the deep lobe of parotid | NA | Surgery | NA | No | No | NA |
| Beltaos and Maurer (1966) | United States of America | Case report | 1 case | M | 42 | Submandibular gland | | Nodule | No | Solitary | Unilateral | NA | No | Surgery | NA | No | No | 8 months- FOD |
| Berkheiser and Clough (1954) | United States of America | Case report | 1 case | F | 53 | Parotid gland | | Nodule | Facial weakness | Solitary | Unilateral | NA | No | Surgery | NA | No | No | 6 months - FOD |
| Bhushan (2023) | India | Case series | 3 cases | M | 30 | Submandibular gland | | Nodule | No | Solitary | Unilateral | CT: heterogeneous enhancing lesion measuring 10 mm | No | Surgery | NA | NA | NA | 24 months - FOD |
|  |  |  |  | F | 63 | Submandibular gland | | Nodule | Pain | Solitary | Unilateral | CT: homogeneous 1.8x1.5 cm enhancing | No | Surgery | NA | Oncocytic hyperplasia | NA | 18 months - FOD |
| Broekhuizen et al. (2011) | Netherlands | Case report | 1 case | NA | 50 | Parotid gland | | NA | NA | Solitary | Unilateral | NA | NA | NA | NA | No | No | NA |
| Buchanan et al. (1988) | United States of America | Case report | 1 case | F | 40 | Nasal septum | | Nodule | Soreness+irritation | Solitary | Unilateral | NA | No | Surgery | NA | No | No | NA |
| Camara et al. (2005) | Brazil | Case report | 1 case | M | 71 | Buccal mucosa | | Nodule | No | Solitary | Unilateral | NA | No | Surgery | NA | No | No | FOD |
| Capo (1965) | United States of America | Case report | 1 case | M | 67 | Larynx | | Nodule | Hoarseness | Solitary | Unilateral | Laminogram shows a mass on middle line above vocal cords | No | Surgery | NA | No | No | 9 months - FOD |
| Chau and Radden (1986) | Australia | Case report | 1 case | M | 58 | Buccal mucosa | | Nodule | No | Solitary | Unilateral | No | No | Surgery | NA | No | No | 30 months - FOD |
| Chaundry and Gorlin (1958) | United States of America | Case report | 1 case | F | 74 | Parotid gland | | Nodule | No | Solitary | Unilateral | No | NA | Surgery | NA | No | No | NA |
| Chen et al. (2016) | United States of America | Case report | 1 case | M | 63 | Submandibular gland | | Nodule | No | Solitary | Unilateral | CT: well-circumscribed mass into the right submandibular space | No | Surgery | No | Oncocytic hyperplasia | No | 12 months - FOD |
| Chui et al. (1985) | United States of America | Case report | 1 case | F | 60 | Ethmoid sinus | | Nodule | Pressure sensation | Solitary | Unilateral | CT: dense mass in the right ethmoid sinus with erosion into the medial and inferior-medial orbital walls | No | Surgery | No | No | No | 36 months - FOD |
| Condington and Carolina (1959) | United States of America | Case report | 1 case | F | 72 | Parotid gland | | Nodule | No | Solitary | Unilateral | NA | No | Surgery | No | No | No | 12 months - FOD |
| Cohen and Batsakis (1968) | United States of America | Case report | 1 case | M | 61 | Nasal cavity | | Nodule | Nasal stuffiness+epistaxis+ rhinorrhea | Solitary | Unilateral | RX: clouded left maxillary antrum and destruction of the superior and medial walls of the left maxillary of the orbit and extension into the malar bone | No | Surgery | NA | No | No | 43 months - FOD |
| Colreavy et al (2001) | Australia | Case report | 1 case | M | 68 | Nasal cavity | | Nodule | Deafness | Multiple | Bilateral | MRI: T-weighted axial scan demonstrating a uniformly enhancing mass filing the post nasal space bilaterally with no skull base invasion | No | Surgery | NA | No | No | 18 months - FOD |
| Cullen et al. (1995) | United States of America | Case report | 1 case | M | 41 | Submandibular gland | | Nodule | No | Solitary | Unilateral | NA | No | Surgery | No | No | Myotonic dystrophy | 2 months -FOD |
| Comin et al. (1997) | Italy | Case report | 1 case | F | 60 | Nasal septum | | Nodule | Epistaxis | Solitary | Unilateral | NA | No | Surgery | NA | No | No | 36 months - FOD |
| Damm et al. (1989) | United States of America | Case report | 1 case | F | 73 | Buccal mucosa | | Nodule | No | Solitary | Unilateral | NA | No | Surgery | NA | No | No | 24 months - FOD |
| Das et al. (1976) | India | Case report | 1 case | F | 7 | Tongue | | Nodule | No | Solitary | Unilateral | NA | No | Surgery | NA | No | No | NA |
| Dastaran and Chandu (2008) | Australia | Case report | 1 case | F | 61 | Submandibular gland | | Nodule | Tenderness | Multiple | Bilateral | CT: 6 mm homogeneously enhancing nodule within the gland. A similar 13 mm nodule in the left submandibular gland with small punctate foci of calcification adjacent to it. | No | Surgery | No | No | Multiple endocrine neoplasia 2B syndrome and neurofibromatosis type 1 | 12 months - FOD |
| Deutsch et al. (1984) | Israel | Case report | 1 case | M | 77 | Parotid gland | | Nodule | Pain | Multiple | Bilateral | NA | NA | No treatment | NA | No | No | NA |
| Dibble and Sanford (1961) | United States of America | Case report | 1 case | M | 79 | Submandibular gland | | Nodule | No | Solitary | Unilateral | NA | NA | Surgery | NA | No | No | NA |
| El Korbi et al. (2019) | Tunisia | Case report | 1 case | M | 62 | Parotid gland | | Nodule | No | Solitary | Unilateral | Ultrasound: hypoechoic vascularized lesion. MRI: hyposignal lesion on T1-weighted imaging and hypersignal on T2-weighted imaging. | No | Surgery | NA | NA | NA | 36 months - FOD |
| Evren et al. (2015) | Turkey | Case report | 1 case | F | 69 | Parotid gland | | Nodule | Pain | Solitary | Unilateral | MRI: 24x16 mm mass of the right superficial parotid lobe superolateral in the largest transverse diameter on fat-suppressed T2-weighted images | No | Surgery | NA | No | Hypercalcemia | 12 months - FOD |
| Farid et al. (2018) | United Kingdom | Case report | 1 case | F | 64 | Nasal septum | | Nodule | Irritation+itch+pain+epistaxis+nasal obstruction | Solitary | Unilateral | NA | No | Surgery | NA | NA | NA | 24 months - FOD |
| Fini et al. (2013) | Italy | Case report | 1 case | F | 70 | Parotid gland | | Nodule | Pain | Solitary | Unilateral | NA | No | Surgery | NA | No | HCV infection and non-Hodgkin lymphoma | 12 months - FOD |
| Ghandur-Mnaymneh (2013) | United States of America | Case report | 1 case | F | 58 | Parotid gland | | Nodule | NA | Solitary | Unilateral | NA | NA | Surgery | NA | No | No | NA |
| Gray et al. (1976) | United States of America | Case series | 10 cases | M | NA | Parotid gland | | Nodule | NA | Solitary | Unilateral | NA | No | NA | NA | NA | NA | NA |
|  |  |  |  | F | 80 | Parotid gland | | Nodule | NA | Solitary | Unilateral | NA | No | NA | NA | NA | NA | NA |
|  |  |  |  | F | 45 | Parotid gland | | Nodule | NA | Solitary | Unilateral | NA | Yes: 156 months | NA | NA | NA | NA | 48 months - FOD |
|  |  |  |  | F | 70 | Parotid gland | | Nodule | NA | Solitary | Unilateral | NA | No | NA | NA | NA | NA | 72 months - FOD |
|  |  |  |  | F | 62 | Parotid gland | | Nodule | NA | Solitary | Unilateral | NA | No | NA | NA | NA | NA | NA |
|  |  |  |  | F | 66 | Parotid gland | | Nodule | NA | Solitary | Unilateral | NA | No | NA | NA | NA | NA | 108 months - FOD |
|  |  |  |  | M | 60 | Parotid gland | | Nodule | NA | Solitary | Unilateral | NA | Yes: 6 months | NA | NA | NA | NA | NA |
|  |  |  |  | M | 77 | Parotid gland | | Nodule | NA | Solitary | Unilateral | NA | No | NA | NA | NA | NA | 12 months - DWD (acute myocardial infarct) |
|  |  |  |  | M | 54 | Parotid gland | | Nodule | NA | Solitary | Unilateral | NA | No | NA | NA | NA | NA | 48 months - FOD |
|  |  |  |  | M | 69 | Parotid gland | | Nodule | NA | Solitary | Unilateral | NA | No | NA | NA | NA | NA | 24 months - FOD |
| Hamada et al. (2018) | Japan | Case report | 1 case | M | 61 | Parotid gland | | Nodule | Pain+facial palsy | Solitary | Unilateral | CT: enhanced irregularly shaped mass. MR: T1-weighted image showing the tumor to have lower intensity than that of the native parotid tissue. T2-weighted image showing the tumor to have an intermediated signal intensity and partial hypersensity. | No | Surgery | NA | NA | NA | 18 months - FOD |
| Hamdan et al. (2002) | United States of America | Case report | 1 case | M | 33 | Nasal septum | | Nodule | Epistaxis | Solitary | Unilateral | NA | NA | Surgery | NA | NA | NA | NA |
| Handler and Ward (1979) | United States of America | Case report | 1 case | M | 64 | Maxillary sinus | | Nodule | Discomfort+paresthesia | Solitary | Unilateral | Sinus X ray: opacification of the left maxillary sinus. CT: mass in left maxillary sinus and erosion of the posterolateral wall. | No | Surgery | NA | NA | NA | 12 months - FOD |
| Hastrup et al. (1982) | Denmark | Case series | 2 cases | F | 69 | Parotid gland | | Nodule | NA | Solitary | Unilateral | NA | Yes: 252 months earlier a tumor had been removed from the same region | Surgery | NA | NA | NA | NA |
|  |  |  |  | F | 55 | Parotid gland | | Nodule | NA | Solitary | Unilateral | NA | NA | Surgery | NA | NA | NA | NA |
| Holmes et al. (1998) | United States of America | Case report | 1 case | M | 71 | Parotid gland | | Nodule | No | Solitary | Unilateral | NA | No | Surgery | NA | NA | NA | NA |
| Hyde (2008) | United States of America | Case report | 1 case | F | 53 | Parotid gland | | Nodule | No | Multiple | Bilateral | MRI: multiple bilateral non-enhancing parotid masses that demonstrated variable intensity on T2 weighting | No | Surgery | NA | Bilateral oncocytic nodular hyperplasia | No | 48 months - FOD |
| Imran et al. (2020) | United States of America | Case report | 1 case | F | 55 | Parotid gland | | Nodule | No | Solitary | Unilateral | CT: 1.9x2.1x1.8 cm circumscribed lesion with heterogeneous enhancement with hypodense region along the anterosuperior portion | NA | Surgery | NA | NA | NA | NA |
| Ito et al. (2000) | Japan | Case series | 2 cases | F | 42 | Parotid gland | | Nodule | No | Solitary | Unilateral | NA | No | Surgery | NA | No | No | 36 months - FOD |
|  |  |  |  | M | 62 | Palate | | Nodule | No | Solitary | Unilateral | NA | No | Surgery | NA | No | NA | 192 months - FOD |
| Jadhav et al. (2017) | India | Case report | 1 case | M | 48 | Parotid gland | | Nodule | No | Solitary | Unilateral | NA | No | Surgery | No | No | No | 48 months - FOD |
| Jalisi (1968) | Pakistan | Case report | 1 case | F | 42 | Parapharyngeal space | | Nodule | Discomfort |  | Unilateral | NA | NA | Surgery | NA | NA | NA | NA |
| Jo et al. (2010) | South Korea | Case series | 5 cases | M | 58 | Parotid gland | | Nodule | NA | Solitary | Unilateral | NA | No | Surgery | NA | No | NA | NA |
|  |  |  |  | M | 47 | Parotid gland | | Nodule | NA | Solitary | Unilateral | NA | No | Surgery | NA | No | NA | NA |
|  |  |  |  | F | 54 | Parotid gland | | Nodule | NA | Solitary | Unilateral | NA | No | Surgery | NA | No | NA | NA |
|  |  |  |  | F | 53 | Parotid gland | | Nodule | NA | Solitary | Unilateral | NA | No | Surgery | NA | No | NA | NA |
|  |  |  |  | F | 56 | Parotid gland | | Nodule | NA | Solitary | Unilateral | NA | No | Surgery | NA | No | NA | NA |
| Johns et al. (1977) | United States of America | Case series | 2 cases | F | 58 | Parotid gland | | Nodule | No | Solitary | Unilateral | NA | No | Surgery | NA | No | NA | 4 months - FOD |
|  |  |  |  | M | 72 | Parotid gland | | Nodule | No | Solitary | Unilateral | NA | No | Surgery | NA | No | NA | 5 months - FOD |
| Kanazawa et al. (2000) | Japan | Case report | 1 case | F | 32 | Buccal mucosa | | Nodule | No | Solitary | Unilateral | CT: distinct solid round mass located in the right cheek | No | Surgery | NA | No | No | 60 months - FOD |
| Kasai et al. (2007) | Japan | Case report | 1 case | F | 56 | Parotid gland | | Nodule | No | Multiple | Bilateral | Ultrasound: multiple rounded hypoechoic masses. MRI: multiple bilateral masses with 10-25 mm in diameter, and lower signal intensities compared to the parotid gland parenchyma on T1-weighted and T2-weighted images. STIR: hard to detect because of similar intensities of the tumor and parenchyma. DW: high intensities on diffusion-weighted images. Dynamic contrast-enhanced MR images showed early enhancement and early washout of the tumors | There is no total excision performed | Surgery | NA | No | NA | 12 months - FOD |
| Kochhar et al. (1990) | India | Case report | 1 case | F | 45 | Palate | | Nodule | No | Solitary | Unilateral | NA | No | Surgery | NA | No | NA | NA |
| Kosuda et al. (1988) | Japan | Case report | 1 case | F | 69 | Parotid gland | | Nodule | No | Solitary | Unilateral | NA | Yes: the paper reports a recurrence 7 months after the surgical resection of the tumor, but the total resection of the primary tumor was not possible, this way, we can't consider a recurrence | Surgery and Iodine-131 therapy in two steps 6 months apart | No | No | No | NA |
| Lane (1962) | United States of America | Case series | 1 case | M | 58 | Parotid gland | | Nodule | No | Solitary | Unilateral | NA | No | Surgery | No | No | NA | 20 months - FOD |
|  |  |  |  | M | 53 | Parotid gland | | Nodule | No | Solitary | Unilateral | NA | NA | Surgery | NA | NA | NA | NA |
| Liu et al. (2000) | United States of America | Case report | 1 case | M | 62 | Parotid gland | | NA | NA | Solitary | Unilateral | NA | No | Surgery | NA | Oncocytosis | Birt-hogg-Dubè syndrome | 12 months - FOD |
| Lopez et al. (2013) | Spain | Case report | 1 case | M | 44 | Multiple: Right nasal cavity, right maxillary sinus, sphenoid, and both sides of the ethmoid sinuses | | Nodule | nasal obstruction+ epistaxis+rhinorrhea | Solitary | Unilateral | CT: solid mass completely involving right nasal cavity, right maxillary sinus, sphenoid, and both sides of the ethmoid sinuses. The planum sphenoidale seemed to be eroded by the tumor. MRI: intradural extension of the tumor and involvement of the cavernous sinus | No | Surgery | NA | No | No | 36 months - FOD |
| Lu et al. (2011) | Taiwan | Case report | 1 case | F | 73 | Parotid gland | | Nodule | No | Solitary | Unilateral | MRI: well-encapsulated mass in the deep lobe of parotid gland. Low signal intensity on T1WI, and isso to slightly hypointensity on short inversion time inversion recovery. | No | Surgery | NA | NA | NA | NA |
| Mair and Johannessen (1970) | Norway | Case serie | 1 case | F | 78 | Parotid gland | | NA | NA | Solitary | Unilateral | NA | No | Surgery | NA | NA | NA | NA |
| Majumdar et al. (2014) | India | Case report | 1 case | F | 53 | Mandible | | Nodule | No | Solitary | Unilateral | Xray: radiopaque lesion | NA | Surgery | NA | NA | NA | NA |
| Matsuki et al. (2021) | Japan | Case report | 1 case | F | 64 | Parotid gland | | Nodule | No | Solitary | Unilateral | MRI: lobulated solid mass with well-defined borders measuring 120x88x60 mm in the deep lobe of the left parotid gland extending into the left parapharyngeal space. The tumor displayed slight hyperintensity on T1-weighted images and hypointensity on T2-weighted images | No | Surgery | No | No | No | 12 months - FOD |
| McLoughlin et al. (1994) | United Kingdom | Case report | 1 case | M | 72 | Submandibular gland | | Nodule | No | Solitary | Unilateral | NA | NA | Surgery | NA | No | No | NA |
| Mercut et al. (2015) | Romania | Case report | 1 case | F | 58 | Parotid gland | | Nodule | Pain | Solitary | Unilateral | Echography: heterogeneous structure. CT: Tumoral mass at the level of the left parotid gland with 28x30x31 mm, well-determined edges and consistency similar to the normal salivary parenchyma | NA | Surgery | NA | No | No | NA |
| Meza-Chavez (1949) | United States of America | Case series | 4 cases | M | 50 | Parotid gland | | Nodule | Bleeding | Solitary | Unilateral | NA | No | Surgical removal + radiotherapy | No | No | No | 16 months - FOD |
|  |  |  |  | F | 72 | Parotid gland | | Nodule | No | Solitary | Unilateral | NA | No | Surgery | NA | No | NA | 152 months - FOD |
|  |  |  |  | F | 60 | Parotid gland | | Nodule | NA | Solitary | Unilateral | NA | NA | Surgery | NA | NA | NA | NA |
|  |  |  |  | M | 32 | Parotid gland | | NA | NA | NA | NA | NA | NA | NA | NA | NA | NA | 132 months - FOD |
| Miracco et al. (1986) | Italy | Case report | 1 case | F | 23 | Nasal cavity | | Nodule | Bleeding | Solitary | Unilateral | NA | No | Surgery | No | No | No | 12 months - FOD |
| Mhapuskar et al. (2011) | India | Case report | 1 case | M | 40 | Parotid gland | | Nodule | No | Solitary | Unilateral | Ultrasonography: well-defined hypoechoic lesion. CT: well-defined isodense soft tissue density lesion in the right parotid gland measuring approximately 2.6x2.9x3.3 cm, without infiltration the adjacent structures | NA | Surgery | No | No | No | NA |
| Motallebnejad et al. (2015) | Iran | Case report | 1 case | M | 36 | Palate | | Nodule | No | Solitary | Unilateral | NA | No | Surgery | No | No | No | 4 months - FOD |
| Murphy et al. (2018) | United Kingdom | Case report | 1 case | M | 76 | Nasal cavity | | Nodule | Epiphora | Solitary | Unilateral | CT: CT performed after the biopsy showed a 7 mm well circumscribed soft tissue swelling in the right inferior meatus | No | Surgery | No | No | NA | unknown - FOD |
| Ozcan et al. (2006) | Turkey | Case report | 1 case | F | 76 | Parotid gland | | NA | No | Solitary | Unilateral | CT: 15x10 mm lesion in the right deep lobe of parotid gland. The lesion had ovoid configuration and homogeneous contrast enhancement. MRI: lesion with 10x15x23 mm in the upper medial side of the deep lobe of right parotid gland with heterogenic contrast, hyperintense in T1 and hypointense in T2 series | No | Surgery | No | Cholesteatoma in the right middle ear. In the ipsilateral parotid gland, there was a multifocal oncocytic hyperplasia | No | 12 months - FOD |
| Palakshappa et al. (2014) | India | Case report | 1 case | F | 32 | Retromandibular region | | Nodule | No | Solitary | Unilateral | NA | NA | Surgery | No | No | No | NA |
| Patil et al. (2012) | United Kingdom | Case report | 1 case | M | 78 | Multiple: Ethmoid and frontal sinuses | | Nodule | Epistaxis | Solitary | Unilateral | CT: opacification of the left ethmoid and frontal sinuses and pneumatization of crista galli. MRI: Left superior meatus involved by the lesion | No | Conservatively follow up annually | No | No | Hairy cell leukemia, hypertension and rheumatoid arthritis | NA |
| Perez et al. (2017) | Spain | Case report | 1 case | M | 74 | Nasopharynx | | Nodule | Hearing loss | Solitary | Unilateral | CT: cystic component nodule | No | Surgery | NA | NA | NA | FOD |
| Popovski et al (2016) | Macedonia | Case report | 1 case | M | 74 | Parotid gland | | Nodule | No | Solitary | Unilateral | MRI: well circumscribed mass in the deep lobe of the right parotid gland with extension into the pharyngeal space | No | Surgery | No | No | NA | 2 months -FOD |
| Ranguelov and Robinson (2003) | United States of America | Case report | 1 case | F | 79 | Parotid gland | | Nodule | Odynophagia+dysphagia | Solitary | Unilateral | CT: large mass in the parotid extending into the parapharyngeal space | NA | Surgery | NA | No | No | NA |
| Rivera and Nelson (2022) | United States of America | Case report | 1 case | F | 63 | Parotid gland | | Nodule | No | Solitary | Unilateral | CT: hyperattenuating lesion within the superficial left parotid gland. MRI: decreased signal in both T1 and T2 weighted images | No | Surgery | NA | No | No | 12 months - FOD |
| Robinson et al. (1990) | United Kingdom | Case report | 1 case | F | 63 | Larynx | | Nodule | Hoarseness+irritation | Solitary | Unilateral | NA | Yes: 3 months and 5 months | Surgery | No | No | No | NA |
| Roden and Levy (1994) | United States of America | Case report | 1 case | F | 73 | Parotid gland | | Nodule | Pain+paresthesia | Solitary | Unilateral | CT: heterogeneous well-circumscribed mass in the posterior aspect of the left parotid gland | No | Surgery | No | No | No | 12 months - FOD |
| Sakai et al. (2003) | Japan | Case report | 1 case | M | 66 | Parotid gland | | NA | No | Solitary | Unilateral | CT: 18x18 mm well-circumscribed mass in the deep lobe of left parotid gland. MRI: well-circumscribed mass in the deep lobe of left parotid gland, with decreased signal intensity on both T1 and T2- weighted images | NA | Surgery | NA | No | NA | NA |
| Sakthikumar et al. (2007) | India | Case report | 1 case | F | 19 | Submandibular gland | | Nodule | Pain | Solitary | Unilateral | CT: encapsulated left submandibular swelling | No | Surgery | NA | No | No | 2 months - FOD |
| Schafer et al. (1956) | United States of America | Case report | 1 case | F | 56 | Parotid gland | | Nodule | No | Solitary | Unilateral | NA | NA | Surgical excision + radiotherapy | No | No | Mild hypertension | NA |
| Sepúlveda et al. (2014) | Chile | Case report | 1 case | M | 67 | Parotid gland | | NA | No | Solitary | Unilateral | CT: isodense expansive mass of 7.3 cm in diameter partially confined to the inside of the left deep parotid lobule with moderate enhancement observed after intravenous contrast media injection. Hypodense areas were present. MRI: large solid expansive process in the deep lobule of the left parotid gland. An isodense area was seen on T1 sequence and mildly hyperintense on T2. The mass crosses the stylomandibular space, parapharyngeal space and oropharyngeal space. Furthermore, it involves the anterior base of tongue and reaches the prevertebral space | No | Surgery | NA | No | No | FOD |
| Shahi et al. (2019) | Nepal | Case report | 1 case | M | 14 | Cheek | | Nodule | No | Solitary | Unilateral | CT: well defined cystic lesion measuring 26x21x19 mm in the right buccal space arising from the buccinator muscle and displacing the zygomaticus major | No | Surgery | NA | No | No | 3 months - FOD |
| Sharma et al. (2018) | India | Case report | 1 case | M | 70 | Parotid gland | | Nodule | No | Solitary | Unilateral | CT: rounded hyperdense lesion in the superficial lobe of parotid gland. | No | Surgery | NA | NA | NA | 24 months - FOD |
| Singh et al. (2023) | India | Case report | 1 case | M | 73 | Parotid gland | | NA | No | Solitary | Unilateral | NA | NA | Surgery | NA | NA | NA | NA |
| Skálová et al. (1999) | Czech Republic | Case serie | 2 cases | F | 73 | Parotid gland | | Nodule | No | Solitary | Unilateral | NA | NA | Surgery | NA | NA | NA | NA |
|  |  |  |  | M | 43 | Parotid gland | | Nodule | No | Multiple | Unilateral | NA | NA | Surgery | NA | NA | NA | NA |
| Stafford et al. (1999) | United States of America | Case report | 1 case | M | 76 | Parotid gland | | Nodule | Facial weakness | Solitary | Unilateral | CT: diffuse enlargement of the right parotid gland | NA | Surgery | NA | No | NA | NA |
| Stomeo et al. (2006) | Italy | Case report | 1 case | F | 67 | Parotid gland | | Nodule | No | Multiple | Bilateral | MRI: egg-shaped mass measuring approximately 2.5 cm in the right right parotid. Left parotid gland: two masses with same characteristics as the right parotid masses | Yes: the left parotid oncocytoma represents a recurrence of an oncocytoma excised 144 months earlier | Surgery | No | No | No | 30 months - FOD |
| Sugiyama et al. (2021) | Japan | Case report | 1 case | F | 54 | Submandibular gland | | NA | No | Solitary | Unilateral | CT: well-circumscribed, round-shaped, homogeneous mass with about 23x24x30 mm | No | Surgery | No | No | No | 30 months - FOD |
| Vlachaki et al. (2009) | Greece | Case report | 1 case | F | 74 | Parotid gland | | Nodule | No | Solitary | Unilateral | CT: egg-shaped mass in the left parotid gland (maximum diameter 3.8 cm) along with cervical lymphadenopathy | No | Surgery | No | No | Refractory immune thrombocytopenic purpura, chronic untreated hepatitis B | 12 months - FOD |
| Watanabe et al. (2011) | Japan | Case report | 1 case | F | 89 | Parotid gland | | Nodule | No | Solitary | Unilateral | MRI: nodule located deeply in the subcutis and attached to the parotid gland | NA | NA | NA | NA | NA | NA |
| Watson et al. (1996) | United States of America | Case report | 1 case | F | 74 | Parotid gland | | NA | No | Multiple | Unilateral | NA | Yes: the current lesion represent a recurrence of a oncocytoma removed 216 months earlier | Surgery | No | Areas of oncocytic hyperplasia | No | 42 months - FOD |
| Wolfowitz et al. (1971) | South Africa | Case report | 1 case | F | 12 | Palate | | Nodule | No | Solitary | Unilateral | NA | NA | Surgery | No | No | No | NA |
| Yaku et al. (1985) | Japan | Case report | 1 case | F | 41 | Nasopharynx | | Nodule | Nasal obstruction | Solitary | Unilateral | Chest X-ray: whiting normal limits | No | Surgery | No | No | No | 24 months - FOD |
| Yamazaki et al. (2018) | Japan | Case report | 1 case | F | 73 | Submandibular gland | | Nodule | No | Solitary | Unilateral | CT: well-defined, oval, homogeneously enhanced mass with 15 mm in diameter in the submandibular gland and some undetected lymph node swelling. MRI: mass with a low signal rim of T2-intensified images | No | Surgery | No | No | No | 12 months - FOD |
| Yilmaz et al. (2011) | Turkey | Case report | 1 case | M | 72 | Alveolar crest | | Nodule | No | Solitary | Unilateral | Rx: tooth radixes which have chronic apical abscess in the related area | NA | Surgery | No | No | No | NA |
| Yoshida et al. (2018) | Japan | Case report | 1 case | F | 44 | Parotid gland | | Nodule | Pain | Solitary | Unilateral | MRI: 35 mm diameter mass in the superficial lobe of the right parotid gland. The lesions were hyperintense on both T1 and T2-weighted imaging. | NA | Surgery | No | No | Birt-Hogg-Dubé Syndrome | NA |
| Yoshihara et al. (1997) | Japan | Case series | 1 case | F | 46 | Parotid gland | | Nodule | No | Solitary | Unilateral | NA | NA | Surgery | No | No | No | NA |
| Zhou and Gao (2009) | China | Case series | 21 cases | F | 49 | Parotid gland | | Nodule | No | Solitary | Unilateral | NA | No | Surgery | No | No | No | 263 months - FOD |
|  |  |  |  | M | 42 | Parotid gland | | Nodule | No | Solitary | Unilateral | NA | No | Surgery | No | No | No | NA |
|  |  |  |  | M | 62 | Parotid gland | | Nodule | No | Solitary | Unilateral | NA | No | Surgery | No | No | No | NA |
|  |  |  |  | M | 6 | Parotid gland | | Nodule | Pain | Solitary | Unilateral | NA | No | Surgery | No | No | No | NA |
|  |  |  |  | M | 72 | Palate | | Nodule | No | Solitary | Unilateral | NA | No | Surgery | No | No | No | 134 months - FOD |
|  |  |  |  | M | 64 | Parotid gland | | Nodule | No | Solitary | Unilateral | NA | No | Surgery | No | No | No | 121 months - FOD |
|  |  |  |  | F | 49 | Parotid gland | | Nodule | No | Solitary | Unilateral | NA | No | Surgery | No | No | No | 96 months - FOD |
|  |  |  |  | M | 61 | Parotid gland | | Nodule | No | Solitary | Unilateral | NA | No | Surgery | No | No | No | 73 months - FOD |
|  |  |  |  | M | 61 | Parotid gland | | Nodule | Pain | Solitary | Unilateral | NA | No | Surgery | No | No | No | 75 months - FOD |
|  |  |  |  | M | 40 | Parotid gland | | Nodule | No | Solitary | Unilateral | NA | No | Surgery | No | No | No | 61 months - FOD |
|  |  |  |  | F | 64 | Parotid gland | | Nodule | Pain | Solitary | Unilateral | NA | No | Surgery | No | No | No | 45 months - FOD |
|  |  |  |  | F | 69 | Parotid gland | | Nodule | No | Solitary | Unilateral | NA | No | Surgery | No | No | No | 51 months - FOD |
|  |  |  |  | M | 81 | Parotid gland | | Nodule | No | Solitary | Unilateral | NA | No | Surgery | No | No | No | 51 months - FOD |
|  |  |  |  | F | 63 | Parotid gland | | Nodule | No | Solitary | Unilateral | NA | No | Surgery | No | No | No | 43 months - FOD |
|  |  |  |  | F | 75 | Parotid gland | | Nodule | Pain | Solitary | Unilateral | NA | No | Surgery and Iodine-125 therapy | No | No | No | 35 months - FOD |
|  |  |  |  | M | 74 | Parotid gland | | Nodule | No | Solitary | Unilateral | NA | No | Surgery | No | No | No | 29 months - FOD |
|  |  |  |  | M | 74 | Parotid gland | | Nodule | No | Solitary | Unilateral | NA | No | Surgery | No | No | No | 27 months - FOD |
|  |  |  |  | F | 49 | Parotid gland | | Nodule | No | Solitary | Unilateral | NA | No | Surgery | No | No | No | 23 months - FOD |
|  |  |  |  | M | 68 | Parotid gland | | Nodule | No | Solitary | Unilateral | NA | No | Surgery | No | No | No | 16 months - FOD |
|  |  |  |  | M | 68 | Parotid gland | | Nodule | No | Solitary | Unilateral | NA | No | Surgery | No | No | No | 9 months - FOD |
|  |  |  |  | M | 72 | Parotid gland | | Nodule | No | Solitary | Unilateral | NA | No | Surgery | No | No | No | 9 months - FOD |
| Ziad et al. (2012) | New Zeland | Case report | 1 case | M | 69 | Parotid gland | | NA | No | Multiple | Unilateral | Ultrasound: enlarged lymph node and multiple other hypoechoic areas in the parotid gland | NA | Surgery | No | Areas of multinodular oncocytosis, multiple oncocytic cysts with intraluminal amylase crystalloids and non-caseating granulomatous inflammation in the excised parotid gland | Asthma, osteoarthritis, gastroesophageal reflux disease and sarcoidosis | NA |
| Ziperman and Capers (1955) | United States of America | Case report | 1 case | F | 5 | Tongue | | Nodule | No | Solitary | Unilateral | NA | No | Surgery | No | No | No | 4 months - FOD |

number, RT: radiotherapy F: female, M: male, NA: not available, CT: computed tomography, MRI: magnetic resonance image, FOD: free of disease, DWD: dead without disease.
