# Supplementary material for: Clinicopathological Study of Oncocytomas of Head and Neck Region: A Systematic Review
Source: J Oral Pathol Med. 2025 Aug 6;54(8):635–46. doi: 10.1111/jop.70022 (PMC12419982; doi:10.1111/jop.70022)
Supplement: Supplementary file 3 — Appendix S3: Histopathological features of oncocytoma cases included in this systematic review. [file JOP-54-635-s002.docx]

| **Author/Year**  **Appendix S3.** Histopathological features of oncocytoma cases included in this systematic review. | **Unilobulated or multilobulated** | **Eosinophilic oncocytes** | **Clear oncocytes** | **Nuclear features** | **Arrangement pattern** | **Cystic spaces** | **Lymphoid stroma** |
| --- | --- | --- | --- | --- | --- | --- | --- |
| Adhikari et al. (2006) | NA | Cells with abundant eosinophilic granular cytoplasm | No | Vesicular nuclei with mild pleomorphism | Solid and trabecular | No | NA |
| Akhtar et al. (2016) | NA | Cells with abundant eosinophilic finely granular cytoplasm | No | Large and located centrally or peripherally and often displayed prominent nucleolus | Solid, sheets, islands and cords | NA | NA |
| Albers et al. (1993) | NA | Cuboidal or cylindrical cells with eosinophilic finely granular cytoplasm | No | Regular round nuclei | Tubular arranged | Yes | NA |
| Altman et al. (1998) | NA | Cells with abundant eosinophilic granular cytoplasm | No | Small and round nuclei | Sheets, nodular, nests and duct-like pattern | Yes | NA |
| Anzalone et al. (2019) | Multilobulated | Cells with granular eoshinophilic cytoplasm | No | Regular round nuclei | Nodular | No | NA |
| Araki and Sakaguchi (2004) | Unilobulated | Cells with abundant granular eosinophilic cytoplasm | No | Uniform and round-to-oval shaped nuclei | Tubular | Yes | NA |
| Askew et al. (1971) | Unilobulated | Large polyhedral cells with eosinophilic granular cytoplasm | No | Dark central nuclei | Solid | No | NA |
| Avila et al. (2019) | Unilobulated | Cells with abundant granular eosinophilic cytoplasm | No | Round to oval vesicular nuclei with prominent nucleoli | Nests, trabeculae, tubules, duct-like structures | Yes | NA |
|  | Unilobulated | Cells with abundant granular eosinophilic cytoplasm | No | Round to oval vesicular nuclei with prominent nucleoli | Nests, trabeculae, tubules, duct-like structures | Yes | NA |
|  | Unilobulated | Cells with abundant granular eosinophilic cytoplasm | Cells with clear cytoplasm and distinct membranes | Round to oval vesicular nuclei with prominent nucleoli | Nests, trabeculae, tubules, duct-like structures | Yes | NA |
|  | Unilobulated | Cells with abundant granular eosinophilic cytoplasm | No | Round to oval vesicular nuclei with prominent nucleoli | Nests, trabeculae, tubules, duct-like structures | Yes | NA |
|  | Unilobulated | Cells with abundant granular eosinophilic cytoplasm | No | Round to oval vesicular nuclei with prominent nucleoli | Nests, trabeculae, tubules, duct-like structures | Yes | NA |
| Banerjee et al. (1995) | Unilobulated | Large cells with abundant eosinophilic granular cytoplasm | No | Small, round and darkly colored nuclei | Solid | No | NA |
| Barrese et al. (2010) | Unilobulated | Cells with prominent eosinophilic granular cytoplasm | No | Round eventually centrally placed dark nuclei | Solid | No | NA |
| Beltaos and Maurer (1966) | Multilobulated | Cells with abundant eosinophilic granular cytoplasm | No | Centrally placed round to oval pale nuclei with prominent nucleoli | Sheets and occasional alveoli of oncocytes separated by thin connective tissue septa | Yes | NA |
| Berkheiser and Clough (1954) | Multilobulated | Large polygonal cells with well-defined borders and acidophilic cytoplasm | No | Small uniform nuclei and usually contained a prominent nucleoli | Cords and duct-like structures | Yes | NA |
| Bhushan (2023) | Multilobulated | Polygonal cells with granular eosinophilic cytoplasm | No | Vesicular nuclei | Duct-like structures and nests, separated by a thin fibrovascular stroma | Yes | No |
|  | Unilobulated | Cells with granular eosinophilic cytoplasm | No | Vesicular nuclei | Nests and clusters | No | NA |
| Broekhuizen et al. (2011) | NA | Polygonal cells with eosinophilic granular cytoplasm | Some of cells showed clear cytoplasm | Round to oval shaped vesicular nuclei with prominent nucleoli | Solid, sheets separated by a thin fibrous septa | Yes | NA |
| Buchanan et al. (1988) | Multilobulated | Polygonal cells with abundant eosinophilic granular cytoplasm | No | Round to oval shaped vesicular nuclei with prominent nucleoli | Areas with solid formation, duct-like structures and sheets separeted by a fibrous septa | Yes | NA |
| Camara et al. (2005) | NA | Eosinophilic cells with abundant granular cytoplasm | No | Hyperchromatic and eosinophilic round to oval nuclei | Solid lobules | NA | NA |
| Capo (1965) | Unilobulated | Collunar cells with eosinophilic granular cytoplasm | No | Round to oval shaped darkly coloured nuclei | Small solid nests, cords with solid with cystic areas | Yes | NA |
| Chau and Radden (1986) | Multilobulated | Columnar to polyhedral epithelial cells with distinct cell boundaries and contained abundant eosinophilic granular cytoplasm | No | Oval vesicular nuclei with occasional prominent nucleoli, and infrequently mitotic figures | Sheets arranged in a alveolar pattern. Solid cords with no apparent duct formation. | No | NA |
| Chaundry and Gorlin (1958) | Multilobulated | Large epithelial cells of different sizes and shapes, and containing abundant eosinophilic granular cytoplasm, with distinct cell boundaries, except where tightly packed. | No | Oval and vesicular nuclei with prominent nucleoli. Some cells have no prominent nucleoli | Sheets separated by a thin fibrous septa | No | NA |
| Chen et al. (2016) | Multilobulated | Epithelial cells with abundant eosinophilic cytoplasm | No | Round nuclei with prominent nucleoli | Solid areas and sheets separated by a thin fibrous septa. Some areas with duct-like formation | Yes | NA |
| Chui et al. (1985) | NA | Large polygonal cells with abundant finely granular eosinophilic cytoplasm | No | Round to oval vesicular nuclei with prominent nucleoli | Cords or alveolar-like clusters separated by a fibrovascular stroma | Yes | NA |
| Condington and Carolina (1959) | Multilobulated | Uniform large cells with abundant pink granular cytoplasm | No | Small round nuclei, some of which were solid, and some were vesicular with prominent nucleoli | Cords, nests, duct-like structures, supported by a scanty conective tissue stroma | Yes | NA |
| Cohen and Batsakis (1968) | NA | Inner columnar and outer cuboidal cells with intensely eosinophilic homogeneous and granular cytoplasm | No | Regular vesicular nuclei with prominent nucleoli | Nests, anastomosing cords, or tubes of epithelial cells supported by a fine fibrovascular stroma | Yes | NA |
| Colreavy et al (2001) | Multilobulated | Large cells with uniformly abundant granular eosinophilic cytoplasm | No | Round small uniformly centrally placed nuclei | Small nests and duct-like structures with central cystic formation, separated by minimal stroma. | Yes | NA |
| Cullen et al. (1995) | NA | Large polyhedral cells with abundant eosinophilic granular cytoplasm | No | Round to oval nuclei, in some cases were vesicular with prominent nucleoli, and other cases, highly hyperchromatic without nucleoli | Solid, nests and duct-like structures | Yes | NA |
| Comin et al. (1997) | Multilobulated | Large oval, cylindrical or polyhedral cells with eosinophilic granular cytoplasm | No | Regular round and vesicular nuclei | Lobes, solid nests, anastomosing cords, trabecular structures with duct-like, showing a small lumina | Yes | NA |
| Damm et al. (1989) | Unilobulated | Polyhedral cells with abundant finely granular eosinophilic cytoplasm | No | Regular round vesicular nuclei with prominent nucleoli | Sheets separated by a fibrovascular septa | No | NA |
| Das et al. (1976) | NA | Polyhedral cells with eosinophilic granular cytoplasm | No | Small round vesicular nuclei with prominent nucleoli | Alveolar, trabecular and duct-like structures separated by delicate fibrous septa | NA | NA |
| Dastaran and Chandu (2008) | NA | Randomly distributed polyhedral cells with abundant eosinophilic granular cytoplasm | No | Mildly enlarged nuclei with central prominent nucleoli, and no significant pleomorphism | Nodules and duct-like structures with intervening fibrosis and calcification in the stroma | Yes | NA |
| Deutsch et al. (1984) | NA | Large polyhedral cells with eosinophilic finely granular cytoplasm | No | Round nuclei | Columns, duct-like structures and occasional tubular formation | Yes | NA |
| Dibble and Sanford (1961) | NA | Polyhedral cels with abundant eosinophilic cytoplasm | No | Round nuclei | Columns, cords and duct-like structures with scant stroma | Yes | NA |
| El Korbi et al. (2019) | Unilobulated | Large polygonal with eosinophilic granular cytoplasm | No | Round vesicular nuclei | Sheets and trabeculae | No | NA |
| Evren et al. (2015) | NA | Large cells with granular eosinophilic circular cytoplasm | No | Moderately marked nuclei, and mild hyperchromatic centrally localized nucleoli | Microfollicular pattern | Yes | NA |
| Farid et al. (2018) | Unilobulated | Large cells with abundant eosinophilic granular cytoplasm | No | Vesicular nuclei | Tubules and duct-like structures | Yes | NA |
| Fini et al. (2013) | Multilobulated | Polyhedral cells with abundant eosinophilic granular cytoplasm | No | Round nuclei | Sheets and occasional duct-like formation | Yes | NA |
| Ghandur-Mnaymneh (2013) | Multilobulated | Large uniform cells with abundant brightly acidophilic cytoplasm | No | Small and uniform nuclei with one or two small nucleoli | Sheets, columns and trabeculae, arranged in nodules separated by fibrous trabeculae that contained residual ducts and acini and cluster of lymphocytes | NA | NA |
| Gray et al. (1976) | Multilobulated | Cells with abundant eosinophilic granular cytoplasm | No | Uniform nuclei with minimal prominent nucleoli | Sheets | No | Yes |
|  | Multilobulated | Cells with abundant eosinophilic granular cytoplasm | No | Uniform nuclei without prominent nucleoli | Solid growth in irregular sheets | No | No |
|  | Unilobulated | Cells with abundant eosinophilic granular cytoplasm | No | Uniform nuclei with marked prominent nucleoli | Focally papillary, cystic mass with fibrous septa | Yes | Yes |
|  | Unilobulated | Cells with abundant eosinophilic granular cytoplasm | No | Uniform nuclei with occasional nucleoli | Focally papillary with prominent duct-like structures | Yes | No |
|  | Multilobulated | Cells with abundant eosinophilic granular cytoplasm and occasional cellular pleomorphism | No | Uniform nuclei with moderate occasional nucleoli | Multinodular growth with prominent fibrous septa | No | No |
|  | Multilobulated | Cells with abundant eosinophilic granular cytoplasm | No | Uniform nuclei with marked prominent nucleoli | Focally papillary | Yes | No |
|  | Unilobulated | Cells with abundant eosinophilic granular cytoplasm | No | Uniform nuclei | Multinodular growth with prominent fibrous septa | No | No |
|  | Unilobulated | Cells with abundant eosinophilic granular cytoplasm and minimal cellular pleomorphism | Cells showed marked clear appearance | Uniform nuclei with prominent nucleoli | Multinodular focally cystic growth | Yes | Yes |
|  | Unilobulated | Cells with abundant eosinophilic granular cytoplasm | Cells showed marked clear appearance | Uniform nuclei with moderate prominent nucleoli | Trabecular pattern with scattered cysts | Yes | Yes |
|  | Unilobulated | Cells with abundant eosinophilic granular cytoplasm | Cells showed marked clear appearance | Uniform nuclei with prominent nucleoli | Predominantly trabecular pattern with cysts | Yes | Yes |
| Hamada et al. (2018) | Multilobulated | No | Clear oncocytes with clear cytoplasm and distinct cellular membranes | Round hyperchromatic and occasionally peripheral nuclei without nucleoli | Sheets, nests and chords of clear oncocytes | NA | NA |
| Hamdan et al. (2002) | Multilobulated | Large epithelial cells with eosinophilic granular cytoplasm | No | Centrally placed bland nuclei | Nests and sheets of oncocytes separated by a thin fibrous septa, and occasionally duct-like structures | Yes | NA |
| Handler and Ward (1979) | NA | Cells with abundant eosinophilic cytoplasm | No | Small and uniform nuclei | Nests, cords and duct-like structures | Yes | NA |
| Hastrup et al. (1982) | Multilobulated | Polyhedral cells with abundant eosinophilic cytoplasm and prominent cell borders | No | Small round and dark nuclei | Tubules and cysts, often with papilliferous projections into the lumens. The epithelium with a single-layered and the tubules and cysts separated by delicate fibrous strands | Yes | NA |
|  | Multilobulated | Polyhedral cells with abundant eosinophilic cytoplasm and prominent cell borders | No | Small round and dark nuclei | Trabeculae, separated by sparse fibrous stroma | NA | NA |
| Holmes et al. (1998) | Unilobulated | Cells with abundant pink granular cytoplasm | No | Uniformly round and centrally-located nuclei with prominent nucleoli | Tightly packed organoid arrangement and occasionally duct-like structures | Yes | NA |
| Hyde (2008) | Multilobulated | Cells with abundant finely granular eosinophilic granular cytoplasm | Focal areas exhibit clear-cell formation | The eosinophilic oncocytes contain a centrally placed round nuclei with prominent nucleoli and slight nuclear pleomorphism. The clear oncocytes had a more peripheral nucleoli | Solid, organoid, and trabecular patterns. Thin fibrous stroma and blood vessels separated the trabecular and organoid forms | No | NA |
| Imran et al. (2020) | Multilobulated | Cells with granular orange-pink cytoplasm | Clear cells streaky with eosinophilic oncocytes | Large single round nuclei with prominent nucleoli | Trabeculae, nests and sheets separated by a thin fibrous septa | No | NA |
| Ito et al. (2000) | NA | Polyhedral or round cells with eosinophilic granular cytoplasm | No | Round nuclei with prominent nucleoli | Trabecular and solid patterns | No | NA |
|  | NA | Polyhedral or round cells with eosinophilic granular cytoplasm | No | Round nuclei with prominent nucleoli | Trabecular and solid patterns | No | NA |
| Jadhav et al. (2017) | Multilobulated | Cells with intensely granular eosinophilic cytoplasm | Occasional cells with clear appearance | Central round vesicular nuclei and indistinct nucleoli | Organoid and trabecular patterns | Yes | NA |
| Jalisi (1968) | NA | Large eosinophilic round to polyhedral cells with granular cytoplasm | No | Some nuclei were round vesicular with prominent nucleoli and others were hyperchromatic with indistinct nucleoli | Nests, trabeculae and solid pattern separated by a thin fibrovascular stroma | Yes | NA |
| Jo et al. (2010) | NA | Large round to polygonal cells with finely granular eosinophilic cytoplasm | No | Central or eccentric round and vesicular nuclei with small but distinct nucleoli | NA | NA | NA |
|  | Multilobulated | Large round to polygonal cells with finely granular eosinophilic cytoplasm | No | Central or eccentric round and vesicular nuclei with small but distinct nucleoli | Cords of oncocytes separated by a thin fibrovascular septa | Yes | NA |
|  | NA | Large round to polygonal cells with finely granular eosinophilic cytoplasm | No | Central or eccentric round and vesicular nuclei with small but distinct nucleoli | NA | NA | NA |
|  | NA | Large round to polygonal cells with finely granular eosinophilic cytoplasm | No | Central or eccentric round and vesicular nuclei with small but distinct nucleoli | NA | NA | NA |
|  | NA | Large round to polygonal cells with finely granular eosinophilic cytoplasm | No | Central or eccentric round and vesicular nuclei with small but distinct nucleoli | NA | NA | NA |
| Johns et al. (1977) | NA | Polyhedral cells with granular eosinophilic cytoplasm | No | Single centrally placed nuclei | NA | NA | NA |
|  | NA | Enlarged eosinophilic cells with granular cytoplasm | Occasional cells with clear appearance | Round vesicular nuclei with distinct nucleoli | Nests and cords of oncocytes | NA | NA |
| Kanazawa et al. (2000) | Unilobulated | Polyhedral cells with abundant granular eosinophilic cytoplasm | No | Centrally located small and round nuclei | Sheets and sparse fibrovascular stroma | NA | NA |
| Kasai et al. (2007) | Unilobulated | Cells with eosinophilic abundant cytoplasm | NA | Small rounded nuclei | NA | NA | NA |
| Kochhar et al. (1990) | Multilobulated | Eosinophilic cells with abundant granular cytoplasm | NA | Vesicular nuclei | Monomorphic sheets of cells separated by thick bands of hyalinised fibro-collagenous tissue | NA | NA |
| Kosuda et al. (1988) | NA | Large cells with eosinophilic granular cytoplasm | No | Round to oval centrally placed nuclei, some of which were vesicular and other pyknotic | Solid sheets | NA | NA |
| Lane (1962) | Multilobulated | Large cells with pale granular eosinophilic cytoplasm | No | Round to oval vesicular nuclei with prominent nucleoli, and few mitotic figures | Solid mass | No | NA |
|  | NA | Cells with eosinophilic granular cytoplasm | No | Pyknotic nuclei | Sheets of oncocytic cells | NA | NA |
| Liu et al. (2000) | NA | Eosinophilic cells with abundant granular cytoplasm | Yes | Vesicular and pyknotic nuclei | Nests and solid | NA | NA |
| Lopez et al. (2013) | NA | Medium-sized cells with abundant finely granular eosinophilic cytoplasm | No | Basophilic vesicular nuclei with prominent nucleoli, moderate pleomorphism, and few mitosis | Solid, trabeculae, sheets, nests, clusters and duct-like structures | Yes | NA |
| Lu et al. (2011) | NA | large eosinophilic granular cells | NA | Small vesicular nuclei | Trabeculae and lobules | NA | NA |
| Mair and Johannessen (1970) | NA | Uniform cells with brightly eosinophilic granular cytoplasm | No | Round to oval centrally placed nuclei | Sheets and occasional alveoli separated by thin connective tissue septa | NA | NA |
| Majumdar et al. (2014) | NA | Eosinophilic granular cells | NA | Vesicular nuclei | Solid and duct-like structures | Yes | NA |
| Matsuki et al. (2021) | Multilobulated | Cells with homogeneous eosinophilic granular cytoplasm | No | Round to oval nuclei. Some of which were vesicular with prominent nucleoli, and others hyperchromatic with inconspicuous nucleoli | Solid sheets and clusters | No | NA |
| McLoughlin et al. (1994) | Multilobulated | Large polyhedral eosinophilic granular cells, and darker spindle-shaped cells | No | Round to oval nuclei | Alveolar pattern with occasional cystic areas. Clusters of oncocytes separated by fibrous septa | Yes | Yes |
| Mercut et al. (2015) | NA | Polygonal cells with well-defined edges, abundant intensely eosinophilic and smoothly granulated cytoplasm | No | Round vesicular nuclei with prominent nucleoli | Solid sheets and islands separated by a thin fibrovascular stroma | NA | NA |
| Meza-Chavez (1949) | Multilobulated | Large epithelial cells, with spherical, oval columnar, pyramidal, or polyhedral shaped with abundant granular highly eosinophilic cytoplasm | No | Small round or oval nuclei with fine granular chromatin evenly distributed and prominent nucleoli, and sometimes two nucleoli | Solid pattern, cords, duct-like structures and tubules | Yes | NA |
|  | Multilobulated | Uniform large polyhedral cells with abundant eosinophilic granular cytoplasm | No | Round centrally placed vesicular nuclei with prominent nucleoli | Solid, clusters, nests separated by a thin fibrovascular stroma, and occasionally duct-like formation | Yes | NA |
|  | Multilobulated | Round, oval, or polygonal cells with finely granular eosinophilic cytoplasm | No | Round centrally placed vesicular nuclei with prominent nucleoli | Clusters and nests separated by a thin fibrovascular stroma | NA | NA |
|  | Multilobulated | Round, oval, or polygonal cells with finely granular eosinophilic cytoplasm | No | Round centrally placed vesicular nuclei with prominent nucleoli | Clusters and nests separated by a thin fibrovascular stroma | NA | NA |
| Miracco et al. (1986) | Multilobulated | Large uniform polyhedral or round cells, with abundant strongly eosinophilic granular cytoplasm | No | Round vesicular nuclei often peripherally placed | Lobules of solid sheets and cords, tubular and duct-like structures, separated by a thin fibrous septa | Yes | Yes |
| Mhapuskar et al. (2011) | NA | Large cells with granular eosinophilic cytoplasm | Some of cells showed clear cytoplasm | Centrally placed vesicular nuclei with prominent nucleoli | Clusters and nests separated by a thin fibrovascular stroma, and occasional duct-like formation | Yes | NA |
| Motallebnejad et al. (2015) | NA | Polyhedral cells with abundant granular eosinophilic cytoplasm | No | Large round vesicular nuclei with prominent nucleoli | Nests and duct-like structures | Yes | NA |
| Murphy et al. (2018) | NA | Round to oval shaped cells with eosinophlic granular cytoplasm | No | Large centrally placed vesicular nuclei with prominent nucleoli | Small, solid nests and duct-like structures with fibrovascular stroma. | Yes | NA |
| Ozcan et al. (2006) | NA | Cells with densely eosinophilic granular cytoplasm | No | Slightly enlarged round to oval and centrally located nuclei with prominent nucleoli | Monotonous sheets with solid trabecular and duct-like structures | Yes | NA |
| Palakshappa et al. (2014) | NA | Polyhedral to round cells with distinct borders and abundant eosinophilic granular cytoplasm | Scattered focal oncocytes with clear appearance | Centrally located round nuclei with little mitotic activity | Solid sheets, alveoli, nests, columns or cords | No | NA |
| Patil et al. (2012) | NA | Large polyhedral cells with abundant eosinophilic granular cytoplasm | No | Uniform round nuclei with prominent nucleoli | Anastomosing trabeculae separated by fibrovascular septa | NA | NA |
| Perez et al. (2017) | NA | Eosinophilic cells with abundant granular cytoplasm | NA | Round to oval vesicular nuclei with prominent nucleoli | Solid, duct-like, clusters | Yes | Yes |
| Popovski et al (2016) | Multilobulated | Large cells with bright eosinophilic cytoplasm | No | Small round vesicular nuclei with prominent nucleoli, or occasional inconspicuous nucleoli | Solid mass with scarce duct-like structures | NA | NA |
| Ranguelov and Robinson (2003) | Multilobulated | Large polygonal to oval cells with finely granular densely eosinophilic cytoplasm | No | Centrally placed round to oval nuclei with dispersed chromatin and small round centrally placed nucleoli | Cords, acini, and duct-like structures | Yes | Yes |
| Rivera and Nelson (2022) | Unilobulated | Large cells with abundant granular eosinophilic cytoplasm | No | Round to oval vesicular nuclei, with single prominent nucleoli | Solid sheets, nests, trabecular separated by thin fibrovascular septa and duct-like structures | No | No |
| Robinson et al. (1990) | Multilobulated | Large cells with abundant strongly eosinophilic cytoplasm | No | Regular small hyperchromatic nuclei | Islands arranged in duct-like structures, with central eosinophilic secretion | Yes | Yes |
| Roden and Levy (1994) | NA | Polyhedral cells with granular eosinophilic cytoplasm | No | Round uniform hyperchromatic nuclei | Solid pattern with large cystic spaces filled with blood | Yes | NA |
| Sakai et al. (2003) | NA | Large cells with abundant eosinophilic granular cytoplasm | No | Small round vesicular nuclei with one or more nucleoli without mitotic figures | Solid clusters and nests separated by a faint stroma in some portions | NA | NA |
| Sakthikumar et al. (2007) | NA | Cells with abundant eosinophilic granular cytoplasm | No | Round to oval vesicular nuclei | Nests and duct-like structures | Yes | NA |
| Schafer et al. (1956) | Multilobulated | Large round or polygonal cells with eosinophilic finely granular cytoplam | No | Small round or oval nuclei with fine chromatin | Solid, cords, columns separated by narrow septa forming a alveolated pattern | NA | Yes |
| Sepúlveda et al. (2014) | NA | Large polygonal cells with abundant eosinophilic granular cytoplasm | No | Round to oval vesicular nuclei with prominent nucleoli | Solid nests and lobules | NA | NA |
| Shahi et al. (2019) | NA | Large polygonal cells with abundant eosinophilic granular cytoplasm | Some of cells showed clear cytoplasm | Round to oval and occasionally polyhedral shaped nuclei with vesicular appearance and prominent nucleoli | Solid islands | NA | NA |
| Sharma et al. (2018) | Unilobulated | Large round cells with abundant granular cytoplasm | No | Round vesicular nuclei | Lobules | NA | NA |
| Singh et al. (2023) | NA | Large polyhedral cell with abundant eosinophilic granular cytoplasm | No | Hyperchromatic nuclei | Sheets | NA | Yes |
| Skálová et al. (1999) | Unilobulated | Cells with abundant eosinophilic granular cytoplasm | No | Vesicular nuclei | Lobules | Yes | NA |
|  | Unilobulated | Cells with abundant eosinophilic granular cytoplasm | Second nodule was composed entirely by clear oncocytes | Vesicular nuclei | Lobules | Yes | NA |
| Stafford et al. (1999) | Unilobulated | Large polygonal cells with abundant eosinophilic granular cytoplasm | No | Round to oval vesicular nuclei with prominent nucleoli | Clusters of oncocytic cells forming tubular structures | Yes | NA |
| Stomeo et al. (2006) | Multilobulated | Polygonal cells with abundant eosinophilic microgranular cytoplasm | Scattered focal oncocytes with clear appearance | Small round vesicular nuclei with prominent nucleoli | Clusters, nests, trabeculae and duct-like structures | Yes | NA |
| Sugiyama et al. (2021) | Unilobulated | Monotonous polygonal cells with abundant eosinophilic granular cytoplasm | No | Round centrally placed vesicular nuclei with prominent nucleoli | Solid clustes and nests | NA | NA |
| Vlachaki et al. (2009) | NA | Epithelial cells with microgranular eosinophilic cytoplasm | No | Small round nuclei | NA | NA | NA |
| Watanabe et al. (2011) | NA | Polygonal cells with abundant eosinophilic microgranular cytoplasm | No | Distinct nuclei with prominent nucleoli | Solid clusters and islands | NA | NA |
| Watson et al. (1996) | Multilobulated | Cells with eosinophilic granular cytoplasm | Some of cells showed clear cytoplasm | Small round to oval nuclei | Lobulated and nodular patterns | NA | NA |
| Wolfowitz et al. (1971) | NA | Polyhedral cells with eosinophilic granular cytoplasm | No | Eccentric vesicular nuclei | Cords, trabeculae and islands | Yes | NA |
| Yaku et al. (1985) | NA | Cells with a finely pale abundant eosinophilic cytoplasm | In some areas clear cells were randomly intermixed with eosinophilic oncocytes | Round and slightly eccentric nuclei with unremarkable nucleoli | Solid nests separated by a fibrous connective stroma | NA | Yes |
| Yamazaki et al. (2018) | Unilobulated | Monotonous epithelial cells with a low N/C ratio and abundant eosinophilic granular cytoplasm | No | Round centrally placed vesicular nuclei with prominent nucleoli | Solid nests and duct-like structures | Yes | NA |
| Yilmaz et al. (2011) | Multilobulated | Polyhedral cells with abundant granular eosinophilic cytoplasm | No | Round vesicular nuclei with prominent nucleoli | Solid sheets | No | NA |
| Yoshida et al. (2018) | Unilobulated | No | Round cells with clear cytoplasm (pale fine granular cytoplasm) | Round centrally or eccentraly placed hyperchromatic nuclei | Solid clusters or trabecular patterns, separated by a thin strands of fibrovascular stroma | No | NA |
| Yoshihara et al. (1997) | NA | Polyhedral cells with finely granular eosinophilic cytoplasm | No | Round to oval centrally placed vesicular nuclei with promiment nucleoli | Solid cords or sheets, alveolar-like clusters with small central lumina | Yes | NA |
| Zhou and Gao (2009) | Unilobulated | Large epithelial cells with eosinophilic granular cytoplasm | No | Round vesicular nuclei | Uniform solid sheets and clusters | No | No |
|  | Unilobulated | Large epithelial cells with eosinophilic granular cytoplasm | No | Round vesicular nuclei | Uniform solid sheets, clusters and duct-like structures | Yes | No |
|  | Unilobulated | Large epithelial cells with eosinophilic granular cytoplasm | No | Round vesicular nuclei | Uniform solid sheets and clusters | No | No |
|  | Unilobulated | Large epithelial cells with eosinophilic granular cytoplasm | Epithelial cells with clear cytoplasm and others with pale eosinophilic granular cytoplasm | Round vesicular nuclei | Uniform solid sheets and clusters | No | No |
|  | Unilobulated | Large epithelial cells with eosinophilic granular cytoplasm | Epithelial cells with clear cytoplasm and others with pale eosinophilic granular cytoplasm | Round vesicular nuclei | Uniform solid sheets and clusters | No | No |
|  | Multilobulated | Large epithelial cells with eosinophilic granular cytoplasm | No | Round vesicular nuclei | Uniform solid sheets and clusters | No | No |
|  | Unilobulated | Large epithelial cells with eosinophilic granular cytoplasm | No | Round vesicular nuclei | Uniform solid sheets and clusters | No | No |
|  | Unilobulated | Large epithelial cells with eosinophilic granular cytoplasm | Epithelial cells with clear cytoplasm and others with pale eosinophilic granular cytoplasm | Round vesicular nuclei | Uniform solid sheets, clusters and duct-like structures | Yes | No |
|  | Multilobulated | Large epithelial cells with eosinophilic granular cytoplasm | No | Round vesicular nuclei | Uniform solid sheets and clusters | No | Yes |
|  | Unilobulated | Large epithelial cells with eosinophilic granular cytoplasm | No | Round vesicular nuclei | Uniform solid sheets, clusters and duct-like structures | Yes | No |
|  | Unilobulated | Large epithelial cells with eosinophilic granular cytoplasm | No | Round vesicular nuclei | Uniform solid sheets and clusters | No | Yes |
|  | Unilobulated | Large epithelial cells with eosinophilic granular cytoplasm | No | Round vesicular nuclei | Uniform solid sheets and clusters | No | No |
|  | Unilobulated | Large epithelial cells with eosinophilic granular cytoplasm | No | Round vesicular nuclei | Uniform solid sheets and clusters | No | No |
|  | Unilobulated | Large epithelial cells with eosinophilic granular cytoplasm | No | Round vesicular nuclei | Uniform solid sheets and clusters | No | No |
|  | NA | Large epithelial cells with eosinophilic granular cytoplasm | No | Round vesicular nuclei | Uniform solid sheets and clusters | No | Yes |
|  | Unilobulated | Large epithelial cells with eosinophilic granular cytoplasm | No | Round vesicular nuclei | Uniform solid sheets and clusters | No | No |
|  | Unilobulated | Large epithelial cells with eosinophilic granular cytoplasm | No | Round vesicular nuclei | Uniform solid sheets, clusters and duct-like structures | Yes | No |
|  | Multilobulated | No | Epithelial cells with clear cytoplasm and others with pale eosinophilic granular cytoplasm | Round to polygonal hyperchromatic nuclei and occasional vesicular nuclei | Uniform solid sheets and clusters | No | Yes |
|  | Unilobulated | Large epithelial cells with eosinophilic granular cytoplasm | No | Round vesicular nuclei | Uniform solid sheets and clusters | No | Yes |
|  | Unilobulated | Large epithelial cells with eosinophilic granular cytoplasm | No | Round vesicular nuclei | Uniform solid sheets, clusters and duct-like structures | Yes | No |
|  | Unilobulated | Large epithelial cells with eosinophilic granular cytoplasm | No | Round vesicular nuclei | Uniform solid sheets, clusters and duct-like structures | Yes | No |
| Ziad et al. (2012) | NA | Cells with abundant eosinophilic granular cytoplasm | No | Round vesicular nuclei | Solid sheets or trabeculae, separated by thin fibrovascular septa | Yes | NA |
| Ziperman and Capers (1955) | NA | Large generally round or oval shaped cells with intense strongly eosinophilic granular cytoplasm | No | Central oval in shape nuclei with prominent nucleoli | Solid clusters of oncocytes and duct-like structures | Yes | NA |

NA: not available
