# Supplementary material for: Clinicopathological Study of Oncocytomas of Head and Neck Region: A Systematic Review
Source: J Oral Pathol Med. 2025 Aug 6;54(8):635–46. doi: 10.1111/jop.70022 (PMC12419982; doi:10.1111/jop.70022)
Supplement: Supplementary file 4 — Appendix S4: Critical appraisal of case reports included in this systematic review. [file JOP-54-635-s003.docx]

**Appendix S4.** Critical appraisal of case reports included in this systematic review.

| **Authors/Year** | **Were patient’s demographic characteristics clearly described?** | **Was the patient’s history clearly described and presented as a timeline?** | **Was the current clinical condition of the patient on presentation clearly described?** | **Were diagnostic tests or assessment methods and the results clearly described?** | **Was the intervention(s) or treatment procedure(s) clearly described?** | **Was the post-intervention clinical condition clearly described?** | **Were adverse events (harms) or unanticipated events identified and described?** | **Does the case report provide takeaway lessons?** | **% of Yes (bias risk)** |
| --- | --- | --- | --- | --- | --- | --- | --- | --- | --- |
| Adhikari et al. (2006) | Y | N | Y | Y | Y | Y | Y | N | 75 (mod) |
| Akhtar et al. (2016) | Y | N | Y | Y | Y | N | N | N | 50 (mod) |
| Albers et al. (1993) | Y | N | Y | Y | Y | Y | N | N | 62.5 (mod) |
| Altman et al. (1998) | Y | Y | Y | Y | Y | Y | Y | N | 87.5 (low) |
| Anzalone et al. (2019) | Y | N | Y | Y | Y | Y | Y | N | 75 (low) |
| Araki and Sakaguchi (2004) | Y | Y | Y | Y | Y | Y | N | N | 75 (low) |
| Askew et al. (1971) | Y | N | Y | Y | Y | N | N | N | 50 (mod) |
| Banerjee et al. (1995) | Y | N | Y | Y | Y | Y | Y | N | 75 (low) |
| Barrese et al. (2010) | Y | Y | Y | Y | Y | N | Y | N | 75 (low) |
| Beltaos and Maurer (1966) | Y | N | Y | Y | Y | Y | N | N | 62.5 (mod) |
| Berkheiser and Clough (1954) | Y | Y | Y | Y | Y | Y | Y | N | 87.5 (low) |
| Broekhuizen et al. (2011) | Y | Y | Y | Y | Y | N | N | N | 62.5 (mod) |
| Buchanan et al. (1988) | Y | N | Y | Y | Y | N | N | Y | 62.5 (mod) |
| Camara et al. (2005) | Y | N | Y | Y | Y | Y | N | N | 62.5 (mod) |
| Capo (1965) | Y | N | Y | Y | Y | Y | Y | N | 75 (low) |
| Chau and Radden (1986) | Y | N | Y | Y | Y | Y | Y | Y | 87.5 (low) |
| Chaundry and Gorlin (1958) | Y | N | Y | Y | Y | N | N | N | 50 (mod) |
| Chen et al. (2016) | Y | N | Y | Y | Y | Y | Y | N | 75 (low) |
| Chui et al. (1985) | Y | Y | Y | Y | Y | Y | Y | Y | 100 (low) |
| Cohen and Batsakis (1968) | Y | N | Y | Y | Y | Y | N | Y | 75 (low) |
| Colreavy et al (2001) | Y | N | Y | Y | Y | Y | N | Y | 75 (low) |
| Comin et al. (1997) | Y | N | Y | Y | Y | Y | N | Y | 75 (low) |
| Condington and Carolina (1959) | Y | Y | Y | Y | Y | Y | Y | Y | 100 (low) |
| Cullen et al. (1995) | Y | N | Y | Y | Y | Y | Y | Y | 87.5 (low) |
| Damm et al. (1989) | Y | N | Y | Y | Y | Y | Y | Y | 87.5 (low) |
| Das et al. (1976) | Y | N | Y | Y | Y | N | Y | N | 62.5 (mod) |
| Dastaran and Chandu (2008) | Y | N | Y | Y | Y | Y | Y | N | 75 (low) |
| Deutsch et al. (1984) | Y | N | Y | Y | Y | Y | N | N | 62.5 (mod) |
| Dibble and Sanford (1961) | Y | Y | Y | Y | Y | N | Y | Y | 87.5 (low) |
| El Korbi et al. (2019) | Y | Y | Y | Y | Y | Y | Y | Y | 100 (low) |
| Evren et al. (2015) | Y | N | Y | Y | Y | Y | N | Y | 75 (low) |
| Farid et al. (2018) | Y | Y | Y | Y | Y | Y | Y | Y | 100 (low) |
| Fini et al. (2013) | Y | Y | Y | Y | Y | Y | Y | Y | 100 (low) |
| Ghandur-Mnaymneh (2013) | Y | N | Y | Y | Y | N | N | N | 50 (mod) |
| Hamada et al. (2018) | Y | Y | Y | Y | Y | Y | Y | Y | 100 (low) |
| Hamdan et al. (2002) | Y | N | Y | Y | Y | N | N | Y | 62.5 (mod) |
| Handler and Ward (1979) | Y | N | Y | Y | Y | Y | Y | Y | 87.5 (low) |
| Holmes et al. (1998) | Y | N | Y | Y | Y | Y | N | Y | 75 (low) |
| Hyde (2008) | Y | N | Y | Y | Y | Y | Y | Y | 87.5 (low) |
| Imran et al. (2020) | Y | Y | Y | Y | Y | N | N | N | 62.5 (mod) |
| Jadhav et al. (2017) | Y | N | Y | Y | Y | Y | N | Y | 75 (low) |
| Jalisi (1968) | Y | Y | Y | Y | Y | N | N | N | 62.5 (mod) |
| Kanazawa et al. (2000) | Y | N | Y | Y | Y | Y | N | N | 62.5 (mod) |
| Kasai et al. (2007) | Y | N | Y | Y | Y | Y | N | N | 62.5 (mod) |
| Kochhar et al. (1990) | Y | N | Y | Y | Y | N | Y | N | 62.5 (mod) |
| Kosuda et al. (1988) | Y | Y | Y | Y | Y | Y | Y | Y | 87.5 (low) |
| Liu et al. (2000) | Y | Y | Y | Y | Y | Y | N | Y | 87.5 (low) |
| Lopez et al. (2013) | Y | Y | Y | Y | Y | Y | Y | Y | 100 (low) |
| Lu et al. (2011) | Y | Y | Y | Y | Y | Y | Y | N | 87.5 (low) |
| Majumdar et al. (2014) | Y | Y | Y | Y | Y | Y | Y | Y | 100 (low) |
| Matsuki et al. (2021) | Y | N | Y | Y | Y | Y | Y | Y | 87.5 (low) |
| McLoughlin et al. (1994) | Y | N | Y | Y | Y | N | Y | Y | 75 (low) |
| Mercut et al. (2015) | Y | N | Y | Y | Y | N | N | Y | 62.5 (mod) |
| Mhapuskar et al. (2011) | Y | N | Y | Y | Y | N | N | Y | 62.5 (mod) |
| Miracco et al. (1986) | Y | N | Y | Y | Y | Y | N | N | 62.5 (mod) |
| Motallebnejad et al. (2015) | Y | N | Y | Y | Y | Y | N | Y | 75 (low) |
| Murphy et al. (2018) | Y | N | Y | Y | Y | Y | Y | Y | 87.5 (low) |
| Ozcan et al. (2006) | Y | N | Y | Y | Y | Y | Y | Y | 87.5 (low) |
| Palakshappa et al. (2014) | Y | N | Y | Y | Y | N | Y | Y | 75 (low) |
| Patil et al. (2012) | Y | N | Y | Y | Y | Y | Y | Y | 87.5 (low) |
| Perez et al. (2017) | Y | Y | Y | Y | Y | N | N | N | 62.5 (mod) |
| Popovski et al (2016) | Y | N | Y | Y | Y | Y | Y | Y | 87.5 (low) |
| Ranguelov and Robinson (2003) | Y | N | Y | Y | Y | N | N | N | 50 (mod) |
| Rivera and Nelson (2022) | Y | Y | Y | Y | Y | Y | N | Y | 87.5 (low) |
| Robinson et al. (1990) | Y | Y | Y | Y | Y | Y | Y | N | 87.5 (low) |
| Roden and Levy (1994) | Y | Y | Y | Y | Y | Y | Y | N | 87.5 (low) |
| Sakai et al. (2003) | Y | N | Y | Y | Y | N | N | N | 50 (mod) |
| Sakthikumar et al. (2007) | Y | N | Y | Y | Y | Y | Y | Y | 87.5 (low) |
| Schafer et al. (1956) | Y | Y | Y | Y | Y | N | N | N | 62.5 (low) |
| Sepúlveda et al. (2014) | Y | N | Y | Y | Y | Y | N | N | 62.5 (low) |
| Shahi et al. (2019) | Y | N | Y | Y | Y | Y | Y | Y | 87.5 (low) |
| Sharma et al. (2018) | Y | N | Y | Y | Y | Y | Y | N | 75 (low) |
| Singh et al. (2023) | Y | Y | Y | Y | Y | N | N | Y | 75 (low) |
| Stafford et al. (1999) | Y | N | Y | Y | Y | N | N | N | 50 (mod) |
| Stomeo et al. (2006) | Y | Y | Y | Y | Y | Y | Y | N | 87.5 (low) |
| Sugiyama et al. (2021) | Y | N | Y | Y | Y | Y | Y | Y | 87.5 (low) |
| Vlachaki et al. (2009) | Y | N | Y | Y | Y | Y | Y | Y | 87.5 (low) |
| Watanabe et al. (2011) | Y | N | Y | Y | Y | N | N | Y | 62.5 (mod) |
| Watson et al. (1996) | Y | N | Y | Y | Y | Y | Y | N | 75 (low) |
| Wolfowitz et al. (1971) | Y | N | Y | Y | Y | N | Y | N | 62.5 (mod) |
| Yaku et al. (1985) | Y | N | Y | Y | Y | Y | N | N | 62.5 (mod) |
| Yamazaki et al. (2018) | Y | N | Y | Y | Y | Y | N | Y | 75 (low) |
| Yilmaz et al. (2011) | Y | N | Y | Y | Y | N | N | N | 50 (mod) |
| Yoshida et al. (2018) | Y | Y | Y | Y | Y | Y | N | Y | 87.5 (low) |
| Ziad et al. (2012) | Y | N | Y | Y | Y | N | N | Y | 62.5 (mod) |
| Ziperman and Capers (1955) | Y | Y | Y | Y | Y | Y | Y | N | 87.5 (low) |

Y: yes, N: No, Mod: moderate.
