# Supplementary material for: Clinicopathological Study of Oncocytomas of Head and Neck Region: A Systematic Review
Source: J Oral Pathol Med. 2025 Aug 6;54(8):635–46. doi: 10.1111/jop.70022 (PMC12419982; doi:10.1111/jop.70022)
Supplement: Supplementary file 5 — Appendix S5: Critical appraisal of case series studies included in this systematic review. [file JOP-54-635-s006.docx]

| Authors | Were there clear criteria for inclusion in the case series? | Was the condition measured in a standard, reliable way for all participants included in the case series? | Were valid methods used for identification of the condition for all participants included in the case series? | Did the case series have consecutive inclusion of participants? | Did the case series have complete inclusion of participants? | Was there clear reporting of the demographics of the participants in the study? | Was there clear reporting of clinical information of the participants? | Were the outcomes or follow-up results of cases clearly reported? | Was there clear reporting of the presenting site(s)/clinic(s) demographic information? | Was statistical analysis appropriate? | % of Yes (risk of bias) |
| --- | --- | --- | --- | --- | --- | --- | --- | --- | --- | --- | --- |
| Avila et al. (2019) | N | Y | Y | N | N | Y | Y | N | Y | N | 50 (mod) |
| Bhushan (2023) | N | Y | Y | N | N | Y | Y | Y | Y | N | 60 (mod) |
| Gray et al. (1976) | Y | Y | Y | N | N | Y | Y | N | Y | N | 60 (mod) |
| Hastrup et al. (1982) | N | Y | Y | Y | Y | Y | Y | N | Y | N | 70 (low) |
| Ito et al. (2000) | N | Y | Y | N | N | Y | Y | N | Y | N | 50 (mod) |
| Jo et al. (2010) | Y | Y | Y | Y | Y | Y | Y | N | Y | Y | 90 (low) |
| Johns et al. (1977) | N | Y | Y | N | N | Y | Y | N | Y | N | 50 (mod) |
| Lane (1962) | N | Y | Y | N | N | Y | Y | Y | Y | N | 60 (mod) |
| Mair and Johannessen (1970) | N | Y | Y | N | N | Y | Y | Y | Y | N | 60 (mod) |
| Meza-Chavez (1949) | N | Y | Y | N | N | Y | Y | Y | Y | N | 60 (mod) |
| Skálová et al. (1999) | N | Y | Y | N | N | Y | Y | N | Y | N | 50 (mod) |
| Yoshihara et al. (1997) | N | Y | Y | N | N | Y | Y | N | Y | N | 50 (mod) |
| Zhou and Gao (2009) | Y | Y | Y | Y | Y | Y | Y | N | Y | N | 80 (low) |
| Y: yes; N: no; Mod: moderate | | | | | | | | | | | |

**Appendix S5.** Critical appraisal of case series studies included in this systematic review.
