# Supplementary material for: Clinicopathological Study of Oncocytomas of Head and Neck Region: A Systematic Review
Source: J Oral Pathol Med. 2025 Aug 6;54(8):635–46. doi: 10.1111/jop.70022 (PMC12419982; doi:10.1111/jop.70022)
Supplement: Supplementary file 6 — Appendix S6: Reference list of studies included in this systematic review but not cited in the text. [file JOP-54-635-s004.docx]

**Appendix S6.** Reference list of studies included in this systematic review but not cited in the text.

1. Adhikari P, Pradhananga RB, Sinha BK, Pradhan B, Thapa N. Oncocytoma of maxillary sinus-a rare presentation. Nepal Med Coll J. 2006; 8(4):292–3.
2. Akhtar K, Qadri S, Ray P, Sherwani R. Oncocytoma of the parotid gland: Dilemma for the cytopathologist. J Orofac Sci. 2016;8(1):66. <https://doi.org/10.4103/0975-8844.181932>.
3. Albers FW, Cuvelier CA, Renders MT, van Cauwenberge PB. Oncocytoma of the nasopharynx. Rhinology. 1993; 31(1):41–3.
4. Altman KW, Cunningham JD, Ainsworth AM. Oncocytoma of the inferior turbinate. J Otolaryngol. 1998; 27(3):176–8.
5. Anzalone CL, Nagelschneider AA, Sims JR, García JJ, DeLone DR, Price DL. Oncocytoma Presenting as a Fat-Containing Intraparotid Mass. Ear Nose Throat J. 2019; 98(7):403–4. <https://doi.org/10.1177/0145561319841210>.
6. Askew JB, Bentinck DC, Jenson AB, Fechner RE. Epithelial and myoepithelial oncocytes. Ultrastructural study of a salivary gland oncocytoma. Arch Otolaryngol. 1971; 93(1):46–54. <https://doi.org/10.1001/archotol.1971.00770060078009>.
7. Avila RE, Samar ME, Fonseca IB, Corball AG, Carriel V, García-Martinez L, Rodríguez I. Oncocytic proliferations of salivary glands: Structural and immunohistochemical study of 7 cases. Int J Odontostomat. 2019;13(1):82. <http://dx.doi.org/10.4067/S0718-381X2019000100082>.
8. Banerjee D, Korzec K, Kim K, Fanelly L. Nasopharyngeal oncocytoma. Otolaryngology - Head and Neck Surgery. 1995; 113(1):136–7. <https://doi.org/10.1016/S0194-5998(95)70157-5>.
9. Beltaos E, Maurer WJ. Oncocytoma of the Submaxillary Salivary Gland: Report of a Case. Archives of Otolaryngology - Head and Neck Surgery. 1966; 84(2):193–7. <https://doi.org/10.1001/archotol.1966.00760030195014>.
10. Berkheiser SW, Clough DM. Oxyphilic adenoma of the parotid gland.Report of a case. Cancer. 1954; 7(4):660–2. [https://doi.org/10.1002/1097-0142(195407)7:4<660::AID-CNCR2820070404>3.0.CO;2-E](https://doi.org/10.1002/1097-0142(195407)7:4%3c660::AID-CNCR2820070404%3e3.0.CO;2-E).
11. Broekhuizen-de Gast H, van Isselt H, Roef M, Lam M. Oncocytoma of the Parotid Gland Causing False-Positive Result on I-131 Whole-Body Scintigraphy. Clin Nucl Med. 2011; 36(8):701–3. <https://doi.org/10.1097/RLU.0b013e318217a65f>.
12. Câmara AC, Kelner N, Kauffman CMF, Lima KP, Henriques ACG, Castro JFL. Oncocytoma of an intraoral minor salivary gland: case report and review of literature. Appl Cancer Res. 2005;25(2):90-2.
13. Capo OA. Oxyphilic Adenoma (Oncocytoma) of the Larynx: Presentation of a Case. Archives of Otolaryngology - Head and Neck Surgery. 1965; 82(1):42–4. <https://doi.org/10.1001/archotol.1965.00760010044010>.
14. Chaudhry AP, Gorlin RJ. Oxyphilic granular cell adenoma (oncocytoma). Oral Surgery, Oral Medicine, Oral Pathology. 1958; 11(8):897–905. <https://doi.org/10.1016/0030-4220(58)90204-4>.
15. Chen B, Hentzelman JI, Walker RJ, Lai J-P. Oncocytoma of the Submandibular Gland: Diagnosis and Treatment Based on Clinicopathology. Case Rep Otolaryngol. 2016; 2016:1–6. <https://doi.org/10.1155/2016/8719030>.
16. Chui RTK, Liao S-Y, Bosworth H. Recurrent Oncocytoma of the Ethmoid Sinus with Orbital Invasion. Otolaryngology–Head and Neck Surgery. 1985; 93(2):267–70. <https://doi.org/10.1177/019459988509300227>.
17. Codington JB, Carolina N. Oxyphilic Granular Cell Adenoma of the Parotid Gland. Am J Surg. 1959; 97(3)333-5. doi:10.1016/0002-9610(59)90312-5.
18. Cohen MA, Batsakis JG. Oncocytic Tumors (Oncocytomas) of Minor Salivary Glands. Archives of Otolaryngology - Head and Neck Surgery. 1968; 88(1):71–3. <https://doi.org/10.1001/archotol.1968.00770010073013>.
19. Comin CE, Dini M, Russo G Lo. Oncocytoma of the nasal cavity: report of a case and review of the literature. J Laryngol Otol. 1997; 111(7):671–3. <https://doi.org/10.1017/S0022215100138290>.
20. Cullen MM, Deutsch MD, Ruhl CM, Bratcher GO. Benign oncocytoma of submandibular gland. Arch Otolaryngol Head Neck Surg. 1995; 121(7):804, 806–7.
21. Damm DD, White DK, Geissler RH, Drummond JF, Henry BB. Benign solid oncocytoma of intraoral minor salivary glands. Oral Surgery, Oral Medicine, Oral Pathology. 1989; 67(1):84–6. <https://doi.org/10.1016/0030-4220(89)90308-3>.
22. Das S, Sengupta P, Chatterjee SK, Sarkar SK. Oncocytoma of tongue in a child. J Pediatr Surg. 1976; 11(1):113–4. <https://doi.org/10.1016/0022-3468(76)90187-1>.
23. Dastaran M, Chandu A. Bilateral submandibular gland oncocytoma in a patient with multiple endocrine neoplasia 2B syndrome and neurofibromatosis type 1: an unusual case. Int J Oral Maxillofac Surg. 2011; 40(7):764–7. https://doi.org/10.1016/j.ijom.2011.02.017.
24. Dibble PA, Sanford DM. Submaxillary Oncocytoma: Oxyphil-Cell Adenoma. Archives of Otolaryngology - Head and Neck Surgery. 1961; 74(3):299–301. <https://doi.org/10.1001/archotol.1961.00740030306011>.
25. Evren C, Demirbilek N, Yiğit VB ilge, Kaur AC emil. Oncocytoma of the parotid gland complicated by hypercalcemia: a case report. Kulak Burun Bogaz Ihtis Derg. 2015; 25(3):179–81. https://doi.org/10.5606/kbbihtisas.2015.02419.
26. Farid M, Protts F, Michael P. Oncocytoma of the nasal septum: A rare cause of persistent irritation. Human Pathology: Case Reports. 2018; 14:60–1. <https://doi.org/10.1016/j.ehpc.2018.08.004>.
27. Fini G, Cascino F, Moricca LM, Scannavivo CM, Mici E, Merola R, et al. Parotid gland oncocytoma in HCV-positive patient with non-Hodgkin’s lymphoma. Case report. G Chir. 2013; 34(1–2):18–20.
28. Ghandur-Mnaymneh L. Multinodular oncocytoma of the parotid gland: A benign lesion simulating malignancy. Hum Pathol. 1984; 15(5):485–6. <https://doi.org/10.1016/S0046-8177(84)80086-6>.
29. Hamdan AL, Kahwagi G, Farhat F, Tawil A. Oncocytoma of the nasal septum: A rare cause of epistaxis. Otolaryngology–Head and Neck Surgery. 2002; 126(4):440–1. <https://doi.org/10.1067/mhn.2002.123925>.
30. Handler SD, Ward PH. Oncocytoma of The Maxillary Sinus. Laryngoscope. 1979; 89(3):372???376. https://doi.org/10.1288/00005537-197903000-00005.
31. Holmes GF, Eisele DW, Rosenthal D, Westra WH. PSA immunoreactivity in a parotid oncocytoma: A diagnostic pitfall in discriminating primary parotid neoplasms from metastatic prostate cancer. Diagn Cytopathol. 1998; 19(3):221–5. [https://doi.org/10.1002/(SICI)1097-0339(199809)19:3<221::AID-DC14>3.0.CO;2-G](https://doi.org/10.1002/(SICI)1097-0339(199809)19:3%3c221::AID-DC14%3e3.0.CO;2-G).
32. Ito K, Tsukuda M, Kawabe R, Kanagawa C, Matsushita K, Kubota A, Madoka F, Kameda Y, Ito T. Benign and Malignant Oncocytoma of the Salivary Glands with Immunohistochemical Evaluation of Ki-67. ORL. 2000; 62(6):338-41.
33. Jadhav M, Haravi R, Kittur S. Oncocytoma of Parotid Gland. Journal of Krishna Institute of Medical Sciences University. 2017; 6(1):109–11.
34. Jalisi M. Oncocytoma of the Accessory Salivary Glands. J Laryngol Otol. 1968; 82(3):257–9. <https://doi.org/10.1017/S0022215100068729>.
35. Jo JH, Choi SH, Roh JL, Nam SY, Kim SY, Cho KJ. Oncocytoma and oncocytic carcinoma of the salivary glands, single institute experience. Korean J Pathol. 2010; 44(4):370–5. <https://doi.org/10.4132/KoreanJPathol.2010.44.4.370>.
36. Johns ME, Regezi JA, Batsakis JG. Oncocytic neoplasms of salivary glands: An ultrastructural study. Laryngoscope. 1977; 87(6):862–71. <https://doi.org/10.1288/00005537-197706000-00002>.
37. Kanazawa H, Furuya T, Murano A, Yamaki M. Oncocytoma of an intraoral minor salivary gland: Report of a case and review of literature. Journal of Oral and Maxillofacial Surgery. 2000; 58(8):894–7. <https://doi.org/10.1053/joms.2000.8217>.
38. Kochhar L, Kumar S, Deka RC, Bose S. Oncocytoma of the minor salivary glands of hard palate. Indian J Otolaryngol. 1990; 42(1):132–3.
39. Kosuda S, Ishikawa M, Tamura K, Mukai M, Kubo A, Hashimoto S. Iodine-131 therapy for parotid oncocytoma. J Nucl Med. 1988; 29(6):1126–9.
40. Lane SL. Oxyphilic granular cell adenoma (oncocytoma) of the parotid. Plast Reconstr Surg. 1962; 30(1):88–94. <https://doi.org/10.1097/00006534-196207000-00010>.
41. Liu V, Kwan T, Page EH. Parotid oncocytoma in the Birt-Hogg-Dube syndrome. J Am Acad Dermatol. 2000; 43(6):1120–2. <https://doi.org/10.1067/mjd.2000.109288>.
42. Mair IWS, Johannessen TA. Benign and malignant oncocytoma of the parotid gland. Laryngoscope. 1972; 82(4):638–42. <https://doi.org/10.1288/00005537-197204000-00009>.
43. Majumdar AB, Paul SS, Sarker G, Ray S. Oncocytoma of Oral Cavity Mimicking as Jaw Tumor. Case Rep Otolaryngol. 2014; 2014:1–5. <https://doi.org/10.1155/2014/315058>.
44. Matsuki T, Tsutsumi S, Miyamoto S, Kano K, Momiyama K, Asako Y, et al. Removal of a Giant Parapharyngeal Space Oncocytoma Without Osteotomy. Ear Nose Throat J. 2021; 103(4):NP203-NP206. <https://doi.org/10.1177/01455613211048973>.
45. McLoughlin P, Barrett A, Speight P. Oncocytoma of the submandibular gland. Int J Oral Maxillofitc Surg. 1994;23:294–5.
46. Mercut V, Iorgulescu D, Popescu S. A case with tumor of left parotid gland and denture stomatitis. Rom J Morphol Embryol. 2015; 56(1):247–50.
47. Meza-Chavez L. Oxyphilic granular cell adenoma of the parotid gland (oncocytoma) report of five cases and study of oxyphilic granular cells (oncocytes) in normal parotid glands. Am J Pathol. 1949;25(3):523–47.
48. Mhapuskar AA, Nagare SP, Chamele J. Oncocytoma of parotid gland: A case report with a review of the literature. Journal of Indian Academy of Oral Medicine and Radiology. 2011:644–6. <https://doi.org/10.5005/jp-journals-10011-1242>.
49. Miracco C, Sensini I, Vessio G, Luzi P. Oncocytic adenoma of the nasal cavity. A case report. Histol Histopathol. 1986; 1(1):9–11.
50. Murphy DC, Alfiky M, Prinsley P. Nasal oncocytoma causing unilateral epiphora in an elderly patient: The vital role of nasendoscopy. BMJ Case Rep. 2018; 2018. <https://doi.org/10.1136/bcr-2017-223183>.
51. Nan-Han L, Chung-Ming K, Jhy-Shyan G, Lee-Ren Y, Yu-Chang L, Chang-Hsien O, et al. Magnetic Resonance Imaging of Parotid Oncocytoma. Journal of Radiological Science. 2011; 36(3):165–8.
52. Özcan C, Talas D, Görür K, Aydin Ö. Incidental deep lobe parotid gland oncocytic neoplasms in an operated larynx cancer patient. Oral Oncology Extra. 2006; 42(6):235–40. <https://doi.org/10.1016/j.ooe.2006.01.003>.
53. Palakshappa SG, Bansal V, Reddy V, Kamarthi N. Oncocytoma of minor salivary gland involving the retromolar region: A rare entity. Journal of Oral and Maxillofacial Pathology. 2014; 18(1):127–30. <https://doi.org/10.4103/0973-029X.131941>.
54. Patil S, Trinidade A, Mercer L, Ashworth P, Lonsdale R, Philpott C. Skull base oncocytoma presenting as epistaxis: An unusual presentation of a rare tumour successfully managed with active surveillance. BMJ Case Rep. 2012. 10:2012:BCR1020115040. <https://doi.org/10.1136/bcr.10.2011.5040>.
55. Pérez SJ, Álvarez CA, Sánchez JDC, Onzono AI. Nasopharyngeal oncocytoma as a cause of eustachian tube dysfunction. Acta Otorrinolaringol Esp. 2017;68(2):124-6.
56. Popovski V, Benedetti A, Monevska DP, Grcev A, Serafimovski P, Pecanovski R, et al. Oncocytoma of the deep lobe of the parotid gland. Macedonian Journal of Medical Sciences. 2016; 4(2):290–2. <https://doi.org/10.3889/oamjms.2016.048>.
57. Ranguelov RD, Robinson RA. Pathologic Quiz Case A 79-Year-Old Woman With an Asymptomatic Oropharyngeal Mass. Arch Pathol Lab Med. 2003; 127(1):e53-55. doi: 10.5858/2003-127-e53-PQC7YO.
58. Robinson AC, Kaberos A, Cox PM, Stearns MP. Oncocytoma of the larynx. J Laryngol Otol. 1990; 104(4):346-9. doi: 10.1017/s0022215100112678.
59. Roden DM, Levy FE. Oncocytoma of the Parotid Gland Presenting with Nerve Paralysis. Otolaryngology–Head and Neck Surgery. 1994; 110(6):587–90. <https://doi.org/10.1177/019459989411000620>.
60. Sakai E, Yoda T, Shinamoto H, Hirano Y, Kusama M, Enomoto S. Pathologic and imaging findings of an oncocytoma in the deep lobe of the parotid gland. Int J Oral Maxillofac Surg. 2003; 32: 563-565.
61. Sepúlveda I, Platín E, Spencer ML, Mucientes P, Frelinghuysen M, Ortega P, et al. Oncocytoma of the parotid gland:a case report and review of the literature. Case Rep Oncol. 2014; 7(1):109–16. <https://doi.org/10.1159/000359998>.
62. Sharma V, Kumar S, Sethi A. Oncocytoma parotid gland. Ann Maxillofac Surg. 2018; 8(2):330–2. <https://doi.org/10.4103/ams.ams_154_17>.
63. Singh J, Chandra A, Srilatha T, Jain T, Raja D, Agrawal R. Oncocytoma of the parotid gland: A rare benign tumour. Journal of Oral and Maxillofacial Pathology. 2023; 27(5):41. <https://doi.org/10.4103/jomfp.jomfp_437_21>.
64. Stafford RE, Ray M, Schubert W. Benign oncocytoma of the deep lobe of the parotid gland. Journal of Oral and Maxillofacial Surgery. 1999; 57(3):346–50. <https://doi.org/10.1016/S0278-2391(99)90688-2>.
65. Sugiyama S, Iwai T, Hirota M, Mitsudo K. Endoscopically-assisted intraoral removal of submandibular gland oncocytoma. J Dent Sci. 2021:561–2. <https://doi.org/10.1016/j.jds.2020.06.017>.
66. Vlachaki E, Tsapas A, Dimitrakopoulos K, Kontzoglou G, Klonizakis I. Parotid gland oncocytoma: A case report. Cases J. 2009; 2(3). <https://doi.org/10.1186/1757-1626-2-6423>.
67. Watanabe T, Yoshida Y, Yamamoto O. Oncocytoma of the parotid gland presenting as a subcutaneous tumor. European Journal of Dermatology. 2011:273–4. <https://doi.org/10.1684/ejd.2010.1246>.
68. Wolfowitz BL, Kotton B, Fernandes C. Oncocytoma of the palate. J Laryngol Otol. 1971; 85(10):1079–82. <https://doi.org/10.1017/S0022215100074491>.
69. Yaku Y, Mori Y, Kanda T, Kaneko T, Kitamura T. Ultrastructural study of glycogen-rich oxyphilic adenoma of the nasopharyngeal minor salivary gland. Virchows Arch A Pathol Anat Histopathol. 1985; 407(2):151–8. <https://doi.org/10.1007/BF00737072>.
70. Yamazaki M, Fukuda M, Nakata A, Nanjo H, Takano H. Solitary oncocytoma of the submandibular salivary gland: A case report. J Oral Maxillofac Surg Med Pathol. 2018; 30(3):281–5. <https://doi.org/10.1016/j.ajoms.2018.02.008>.
71. Yilmaz N, Bereket M, Temiz P. An uncommon benign tumor of oral minor salivary glands: a case of oncocytoma. J Dent Fac Atatürk Üniv. 2011; 4(1):77–9.
72. Yoshihara T, Satoh M, Yamamura Y, Yaku Y. An ultrastructural study of oncocytoma and oncocytic carcinoma of the parotid gland. Medical Electron Microscopy. 1997; 30(1):31–6. <https://doi.org/10.1007/BF01458349>.
73. Ziperman H, Capers T. Oxyphil cell adenoma of the tongue. US Armed Forces Med J. 1955; 6(7):1039–42.
